# Sperm-specific histone H1 in highly condensed sperm nucleus of *Sargassum horneri*

**DOI:** 10.1038/s41598-024-53729-2

**Published:** 2024-02-09

**Authors:** Yu Takeuchi, Shinya Sato, Chikako Nagasato, Taizo Motomura, Shujiro Okuda, Masahiro Kasahara, Fumio Takahashi, Shinya Yoshikawa

**Affiliations:** 1 Faculty of Marine Science and Technology, Fukui Prefectural University, 1-1 Gakuencho, Obama, Fukui 917-0003 Japan; 2 Field Science Center for Northern Biosphere, Muroran Marine Station, Hokkaido University, Muroran, 051-0013 Japan; 3 Graduate School of Medical and Dental Science, Niigata University, 1-757 Asahimachi, Chuoku, Niigata, Niigata 951-8501 Japan; 4 Graduate School of Life Sciences, Ritsumeikan University, 1-1-1 Noji-Higashi, Kusatsu, Shiga 525-8577 Japan; 5 Faculty of Pharmaceutical Sciences, Toho University, Funabashi, Chiba 274-8510 Japan

**Keywords:** Plant morphogenesis, Evolutionary developmental biology, Spermatogenesis

## Abstract

Spermatogenesis is one of the most dramatic changes in cell differentiation. Remarkable chromatin condensation of the nucleus is observed in animal, plant, and algal sperm. Sperm nuclear basic proteins (SNBPs), such as protamine and sperm-specific histone, are involved in chromatin condensation of the sperm nucleus. Among brown algae, sperm of the oogamous Fucales algae have a condensed nucleus. However, the existence of sperm-specific SNBPs in Fucales algae was unclear. Here, we identified linker histone (histone H1) proteins in the sperm and analyzed changes in their gene expression pattern during spermatogenesis in *Sargassum horneri*. A search of transcriptomic data for *histone H1* genes in showed six histone H1 genes, which we named *ShH1.1a*, *ShH1b*, *ShH1.2*, *ShH1.3*, *ShH1.4*, and *ShH1.5*. Analysis of SNBPs using SDS-PAGE and LC–MS/MS showed that sperm nuclei contain histone ShH1.2, ShH1.3, and ShH1.4 in addition to core histones. Both *ShH1.2* and *ShH1.3* genes were expressed in the vegetative thallus and the male and female receptacles (the organs producing antheridium or oogonium). Meanwhile, the *ShH1.4* gene was expressed in the male receptacle but not in the vegetative thallus and female receptacles. From these results, *ShH1.4* may be a sperm-specific histone H1 of *S. horneri.*

## Introduction

Spermatogenesis, involving chromatin condensation, reduction of cytoplasm volume, and flagellum formation, is one of the most drastic cell differentiation processes and makes sperm fertile. In particular, chromatin condensation of the sperm nucleus affects sperm motility and fertilization rate^1–3^. Interestingly, sperm nucleus condensation has been observed in animals^4–6^, some land plants^7,8^, and algae^9–12^.

In somatic cells, chromatin is composed of nucleosomes, which are DNA wrapped around histone octamers, including two copies of histones H2A, H2B, H3, and H4. Each nucleosome is linked to histone H1. Chromatin is generally restructured with sperm nuclear basic proteins (SNBPs) such as protamine, protamine-like protein, and sperm-specific histone, and SNBPs induce chromatin condensation in the sperm nucleus^13,14^. The diversity and molecular mechanisms behind chromatin condensation in sperm have mainly been studied in animals, but little is known about them in plants and algae.

SNBPs are classified into three types: protamine (P)-type, histone (H)-type, and protamine-like protein (PL)-type. Protamine types are sperm in which the SNBPs are mostly protamine with low molecular weight and high arginine content, which have been reported in many animals, e.g., humans^15^ and salmon^16^. In mammalian spermiogenesis, most of the histones that compose the nucleosome disappear, and chromatin remodeling occurs with the appearance of protamine through the transition protein^17^. H-types have been well reported in marine invertebrates, sea urchin^18^, starfish^19^, and sponge^20^, among others. Among H-types, sperm-specific H1 histones have been identified in several species^18–21^. Because the sperm-specific H1 histones function in compacting and stabilizing^20^, the replacement of somatic-type H1 with sperm-specific H1 histone(s) may be associated with significant chromatin condensation in the sperm nucleus of H-type. Protamine-like proteins have properties that are intermediate between those of protamine and histone H1 in terms of their structure and amino acid composition, suggesting that protamine evolved through the PL-type from histone H1 in animals^13,14,22^.

Besides the findings in animals, Reynolds and Wolfe (1984) reported that protamine proteins were detected in sperm of Charophyta (*Chara corallina*), bryophyte (*Marchantia polymorpha*), and fern (*Marsilea vestitia*)^23^. Recent studies also showed a protamine-like gene expressed in the sperm of the bryophyte *Marchantia polymorpha*^24,25^*.*

Brown algae are multicellular photosynthetic organisms that belong to the Heterokontophyta. During sexual reproduction, swimming gametes form. In brown algae, three types of sexual reproduction, isogamy, anisogamy, and oogamy, are observed^26^. Sperm nuclear condensation is observed in oogamous brown algae^10–12^, as in animals. In particular, it has been reported that sperm of the Fucales are markedly condensed during spermatogenesis^27^ and that the SNBP of their spermatozoa is of the H-type^28,29^. Elucidation of the condensation mechanism in sperm nuclei of brown algae, which are phylogenetically distinct from the animals and land plants, may contribute to understanding the universality and diversity of sperm. Previously, we showed that the band pattern of histone H1 proteins in SDS-PAGE differed between somatic cells and sperm nuclei in *Sargassum confusum*^29^. This suggests that some sperm-specific H1 histone(s) may be involved in the condensation of sperm nuclei in *S. confusum*. However, there is no clear evidence that sperm-specific *histone H1* genes exist in *Sargassum*.

Histone H1 consists of an N-terminal domain (NTD) and a carboxy-terminal domain (CTD) flanking a globular domain (GD) that binds to the nucleosome core. The GD and CD are relatively highly conserved, whereas the NTD is more poorly conserved. Histone H1 generally forms higher-order chromatin structures and regulates gene expression^30^. Biochemical analyses have reported that the green alga *Chlamydomonas reinhardtii* contains two histone H1 genes^31^, genome sequencing has shown that the brown alga *Ectocarpus siliculosus* contains nine *histone H1* genes^32^ and the red alga *Cyanidioschyzon merolae* contains one^33^. Although in *Arabidopsis thaliana*, histone H1 is reportedly required for heterochromatin condensation^34^, the histone H1 variant, which is thought to be involved in the nuclear condensation of male gametophytes in plants and algae, has not been reported. In the present study, *Sargassum horneri*, which is closely related to *S. confusum* and for which culture strains have been established, was used to investigate the presence or absence of sperm-specific histone H1. First, RNA-seq data (PRJDB4109)^35^ of thallus with reproductive organ (receptacle) was used to identify the *histone H1* gene in the *S. horneri* genome. Next, one of the histone H1 proteins in the sperm nucleus was shown to be expressed in the male reproductive tissue, the male receptacle, but not in the vegetative tissue and female receptacles. Finally, to elucidate the evolution of histone H1 in brown algae, we performed a phylogenetic analysis of histone H1s in brown algae, adding new data on histone H1 in three species to the previously reported genomic and transcriptomic data of brown algae.

## Results

### Spermatogenesis of *S. horneri*

**Figure 1:**
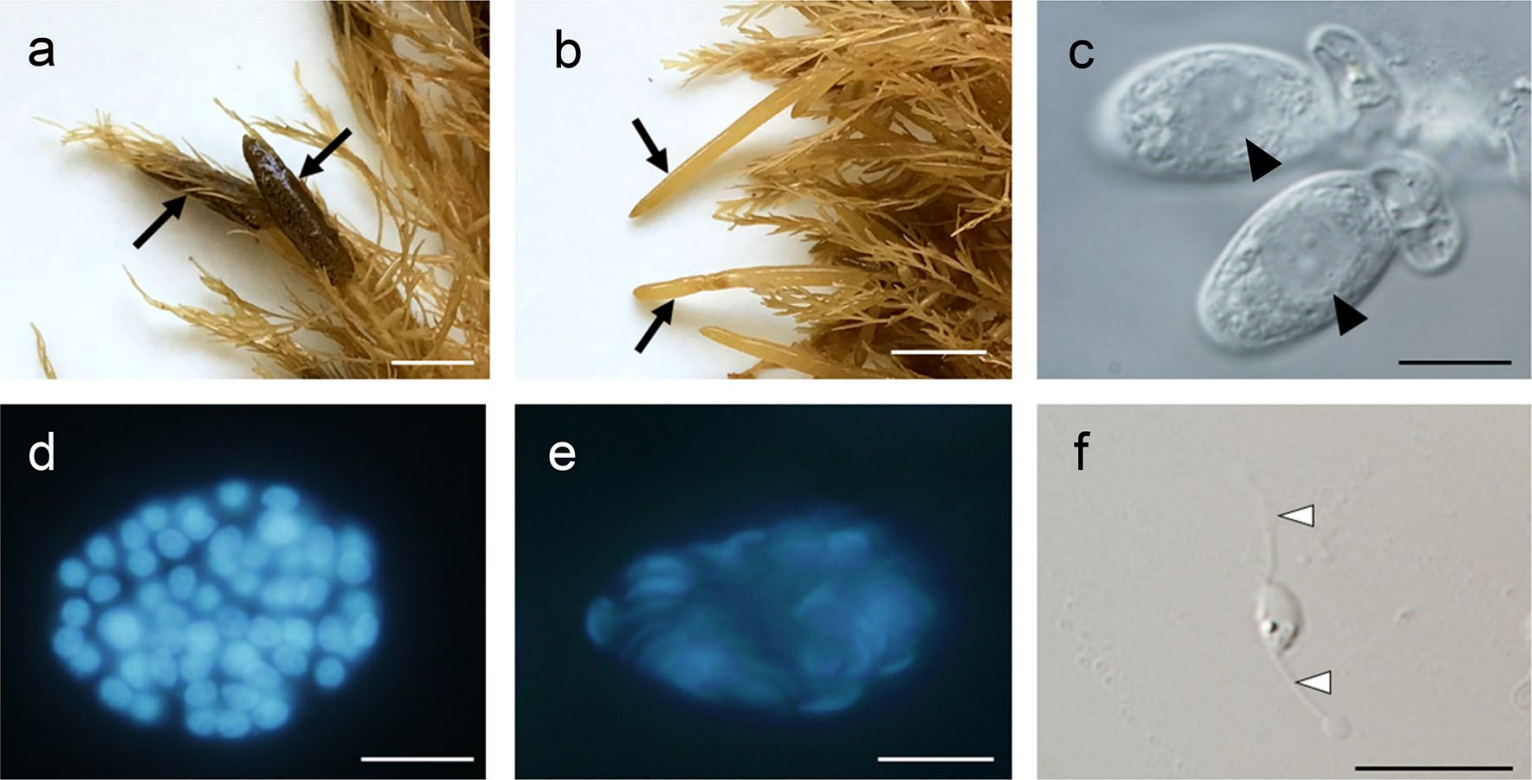
The receptacles, antheridia, and sperm of *Sargassum horneri*. Female (**a**) and male (**b**) receptacles. Arrows indicate mature receptacles. One-nucleus-stage antheridium in bright field (**c**). Arrowheads indicate the nucleus. Fluorescence microscopy observation of antheridum. Antheridium of 64-nuclei-stage nuclei before (**d**) and after chromatin condensation (**e**). The nuclei of antheridium were stained with DAPI. Sperm (**f**). White arrowheads indicate flagella. Scale bars, 1 cm (**a**,**b**), 10 μm (**c**–**f**).

In Wakasa-cho, Fukui, in the middle of Japan’s Sea of Japan coastline, the thallus of *S. horneri* was matured, namely, receptacles were formed in April and May. Male and female thalli were distinguished by the shape of the receptacles (Fig. 1a and b). Male receptacles have an elongated shape compared with female receptacles. The onset of spermatogenesis generally coincided with the day of spring tide. After nuclei in the antheridium increased from 1 nucleus (1-nucleus stage) to 64 nuclei (64-nuclei stage), the sperm nuclei condensed and sperm with two flagella were released (Fig. 1c–f). Using fluorescence and electron microscopy, we have previously observed the nuclear condensation process during sperm formation in *Stephanocystis hakodatensis* (*former Cystoseira hakodatensis*) of the order Fucales. We reported that nuclear condensation occurs after the division of up to 64 nuclei.

In this study, a similar pattern was observed during sperm formation in *S. horneri*, where nuclear condensation did not occur in the 1-nucleus stage but was confirmed to take place after the division of up to 64 nuclei within the antheridium.

### Identification of *histone H1* gene in *S. horneri*

**Figure 2:**
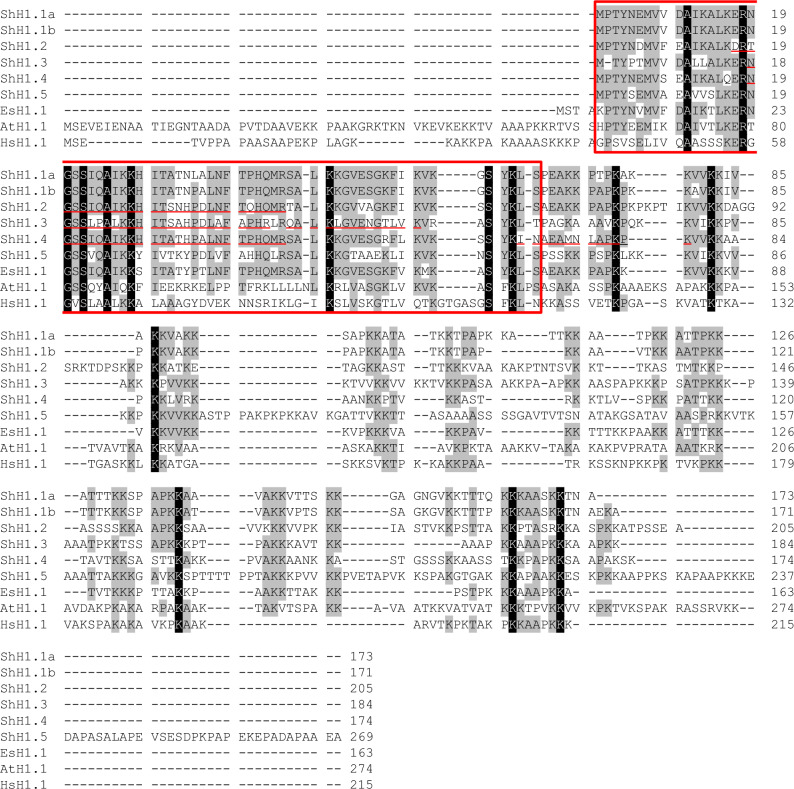
Amino acid sequence alignment of histone H1s of *Sargassum horneri* (ShH1.1a–ShH1.5), *Ectocarpus siliculosus* (EcH1.1, accession number: CBJ32074), *Arabidopsis thaliana* (AtH1, accession number: AAF63139), and *Homo sapiens* (HsH1, accession number: NP_005316.1). A red box indicates the globular domain. Amino acid residues that are majority identical (> 50%) and exactly identical (= 100%) are shown in gray and black, respectively. Red underlines represent amino acids detected by LC–MS/MS.

Previously, we obtained transcriptomic data from *S. horneri* (PRJDB4109)^28^. The full lengths of six histone H1 sequences, *ShH1.1a* (LC765405), *Sh1.1b* (LC765406), *ShH1.2* (LC765407), *ShH1.3* (LC765408), *ShH1.4* (LC765409), and *ShH1.5* (LC765410), were identified from the transcriptomic data (Table 1, Fig. 2). Amino acid sequences of *ShH1.1a* and *ShH1.1b* were highly similar (Supplementary Table S1). The predicted molecular mass of *Sh H1.5* was the largest among histone ShH1s.

**Table 1:** Characteristics of histone H1s in *Sargassum horneri*.

Histones	Accession number	Predicted molecular mass	Lysine and Arginine contents in the carboxy-terminal domain	
			Lysine (%)	Arginine (%)
ShH1.1a	LC765405	18.4	35.5	0.0
ShH1.1b	LC765406	18.2	37.1	0.0
ShH1.2	LC765407	21.9	31.7	1.4
ShH1.3	LC765408	19.4	37.8	0.0
ShH1.4	LC765409	18.6	31.5	1.9
ShH1.5	LC765410	27.8	26.1	0.5

The proportions of basic amino acids in the CTD involved in the binding of histone H1 to DNA^30^ are shown in Table 1. As previously reported, the proportion of lysine residues was high in all six histone ShH1s. The rate of lysine residues peaked in ShH1.3 (37.8%) and reached its nadir in ShH1.5 (26.1%). All six ShH1s had low contents of arginine residues; the highest rate was for ShH1.2 (1.4%), while ShH1.1a, ShH1.1b, and ShH1.3 did not contain any arginine residues in their CTD.

Histone H1s of *S. horneri* were aligned with histone H1s of *E. siliculosus* (EcH1.1), *Homo sapiens* (HsH1.1), and *Arabidopsis thaliana* (AtH1.1) (Fig. 2 and Supplementary Fig. 1). In general, histone H1s, for example HsH1.1 and AtH1.1, are composed of an NTD, GD, and CTD^30^. However, no histone H1s of *S. horneri* have an NTD. As in *S. horneri*, the histone H1s of *E. siliculosus* either started in the GD or only a few bases were attached to the GD (Supplementary Fig. S1).

The GD is well conserved among the histone H1s (Fig. 2 and Supplementary Table S1). The pairwise identity of identical residues in the GD between histone H1 of *S. horneri* was a minimum of 55.2% (ShH1.3 and ShH1.5) and a maximum of 98.5% (ShH1.1a and ShH1.1b). Comparing the GDs of histone H1s in *S. horneri* to those in *E. siliculosus*, *Arabidopsis thaliana*, and *Homo sapiens*, sequence identities were 58.2–85.1%, 42.0–50.7%, and 23.6–33.3%, respectively.

### Identification of histone H1 among basic proteins of *S. horneri* sperm nuclei

**Figure 3:**
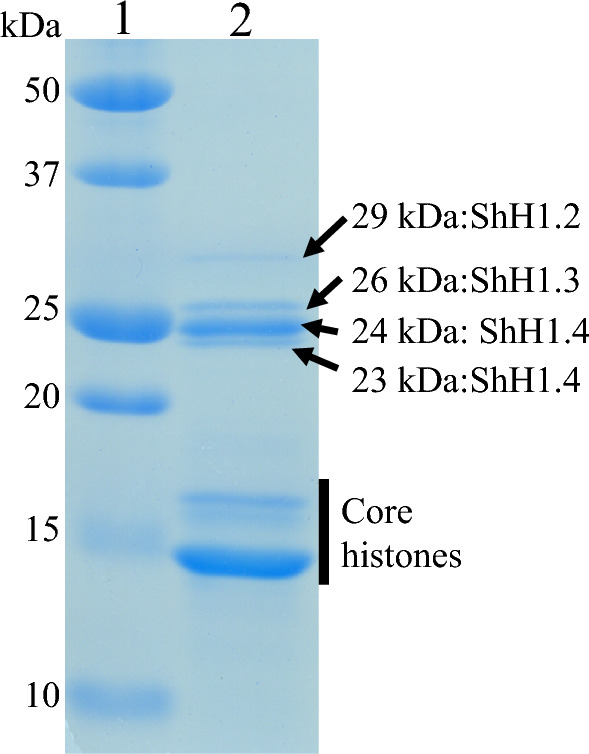
SDS-PAGE analysis of basic proteins extracted from sperm nuclei of *Sargassum horneri*. Line 1: molecular markers, line 2: basic proteins extracted from sperm nuclei of *Sargassum horneri*. Arrows indicate histone H1 and core histones are shown by the bar.

SNBPs of *S. horneri* were analyzed by SDS-PAGE and LC–MS/MS (Figs. 2 and 3). Four bands around 25 kDa (arrows) were predicted to represent histone H1, while three bands between 20 and 15 kDa were predicted to represent core histones. The band pattern of core histones was similar to those of Sargassaceae algae, *Stephanocystis hakodatensis* and *Sargassum confusum*^28,29^. Predicted histone H1s, with apparent molecular masses of 29, 26, 24, and 23 kDa, were identified by LC–MS/MS and transcriptomic data of *S. horneri* (Figs. 2 and 3). Proteins were identified by A2 values using the Mascot search engine (See supplementary LC–MS/MS data). Except for the band with an apparent molecular mass of 29 kDa, we identified the protein with the highest A2 value. The apparent 29 kDa molecular mass band had the highest A2 value of 12.38 for the hypothetical protein. However, as the predicted molecular mass of the putative protein was 58.8 kDa, the 29 kDa protein was identified as histone H1.2, which had the second-highest A2 value (12.29). The peptide sequence obtained from the bands of apparent molecular masses 26 kDa was consistent with the amino acid sequences of histone ShH1.3. Both of the peptide sequences obtained from the other two bands, of 24 and 23 kDa, matched histone ShH1.4. This suggested that histone H1s of both 24 and 23 kDa were transcripts of the *histone H1.4* gene. The apparent difference in molecular weight is probably due to post-translational modifications of ShH1.4, for example, phosphorylation and post-translational cleavage. (The band of an apparent molecular mass of 23 kDa is referred to as H1.4’.) The four ShH1 proteins were also separated by two-dimensional electrophoresis (Supplementary Fig. S2).

Gene expression analysis of *histone H1s* contained in *S. horneri* sperm nuclei.

To determine whether the three histone H1s present in sperm were expressed in a sperm-specific manner, we analyzed the expression levels of *histone ShH1* genes in the vegetative thalli, the female receptacles that contain oogonium, and the male receptacles possessing antheridium with condensed sperm nuclei (Fig. 1a, b, and e), using reverse-transcription (RT)-PCR. We also analyzed the expression of the sperm-specific mastigoneme-related protein gene (*ShMRP*). Mastigonemes are structures that are only observed in flagellated cells; therefore, in this study, *ShMRP* was used as an indicator of sperm-specific gene expression^36,37^.

**Figure 4:**
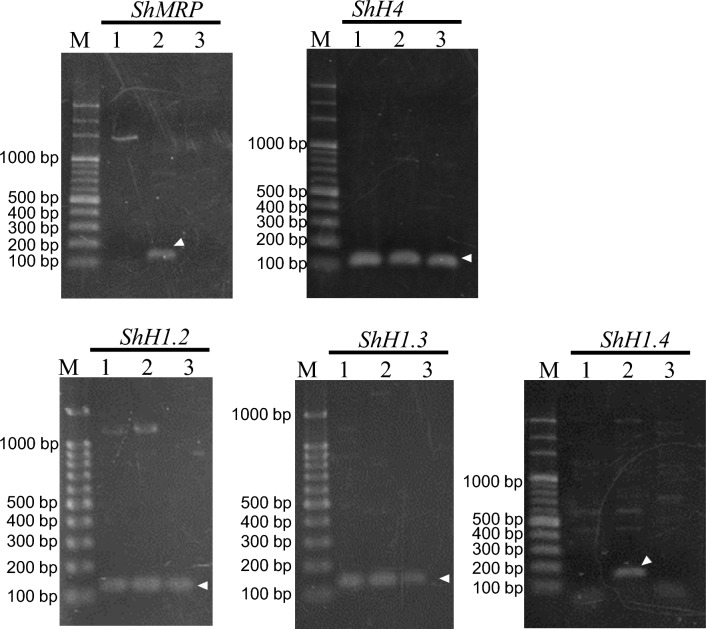
Gene expression analysis of *ShH1.2*, *Sh1.3*, *Sh1.4*, *histone ShH4*, and *mastigoneme-related protein (ShMRP)* in the vegetative thallus, and male and female receptacles of *Sargassum horneri* by RT-PCR. *histone H4* genes were used as positive controls. *ShMRP* gene is used as a gene specifically expressed in sperm. An arrowhead indicates the product of the desired amplification. M: DNA 100 bp ladder marker. Lane 1: vegetative thallus, lane 2: male receptacles, and lane 3: female receptacles.

The size of the target amplification products were 100–200 bp for all genes (Supplement Table S2). For some genes, bands of more than 500 bp were detected in addition to the expected amplified product, which appeared to be non-specific amplification. The expression profile of *ShH1.4* was confirmed only in male receptacles, which was the same result as for *ShMRP* (Fig. 4). Comparison of the expression levels of *ShH1.2, 3* and *4* in the vegetative thallus by real-time PCR showed that *Sh1.4* was significantly less expressed than the other histones (Supplementary Fig. S3). *ShH1.2* and *3* were expressed in the vegetative thallus and male and female receptacles, as well as in *histone H4 gene* (ShH4), which were used as controls. (Fig. 4). The expression of three *histone H1* genes, *ShH1.2a*, *ShH1.1b*, and *ShH1.5*, whose proteins were not detected in sperm nuclei by SDS-PAGE, was also examined. RT-PCR analysis showed that *ShH1.1a*, *ShH1.b*, and *ShH1.5* were expressed in the vegetative thallus (Supplementary Fig. S4).

**Figure 5:**
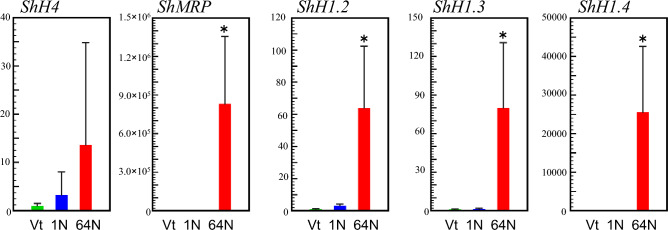
*ShH1.2*, *ShH1.3*, *ShH1.4, ShH4* and ShMPR gene expression analysis in vegetative thallus (Vt), and 1- (1N) and 64-nuclei-stage (64N) conceptacles containing male receptacles by real-time RCR. The relative expression of each gene in Vt is set to 1. The *actin* gene is used as an internal control. A bar represents the standard deviation, while an asterisk indicates *P* < 0.01 (n = 5).

Furthermore, gene expression of histone H1s was analyzed by quantitative RT-PCR during the spermatogenesis of *S. horneri* (Fig. 5). Detailed results of quantitative RT-PCR are presented in the Supplementary Table S3. The gene expression levels were investigated and compared between vegetative thalli, and receptacles containing 1-nucleus-stage (Fig. 1c) and 64-nuclei-stage (Fig. 1e) antheridia. The expression levels of *ShH1.2*, *ShH1.3*, *ShH1.4*, and *ShMRP* were significantly increased in the receptacles containing 64-nuclei-stage antheridia (P < 0.01). Although the expression levels of core histone *ShH4* were also elevated, this was not significant. The rates of increase of *MRP*, *ShH1.2*, *ShH1.3*, and *ShH1.4* was 832,249-, 64-, 80-, and 25,549-fold, respectively. The fold change of *ShH1.4* gene expression in receptacles with condensed-nuclei-stage antheridia was bigger than that of *ShH1.2* and *ShH1.3* and comparable to that of *ShMRP*.

### Phylogenetic analysis of histone H1 protein in brown algae

**Figure 6:**
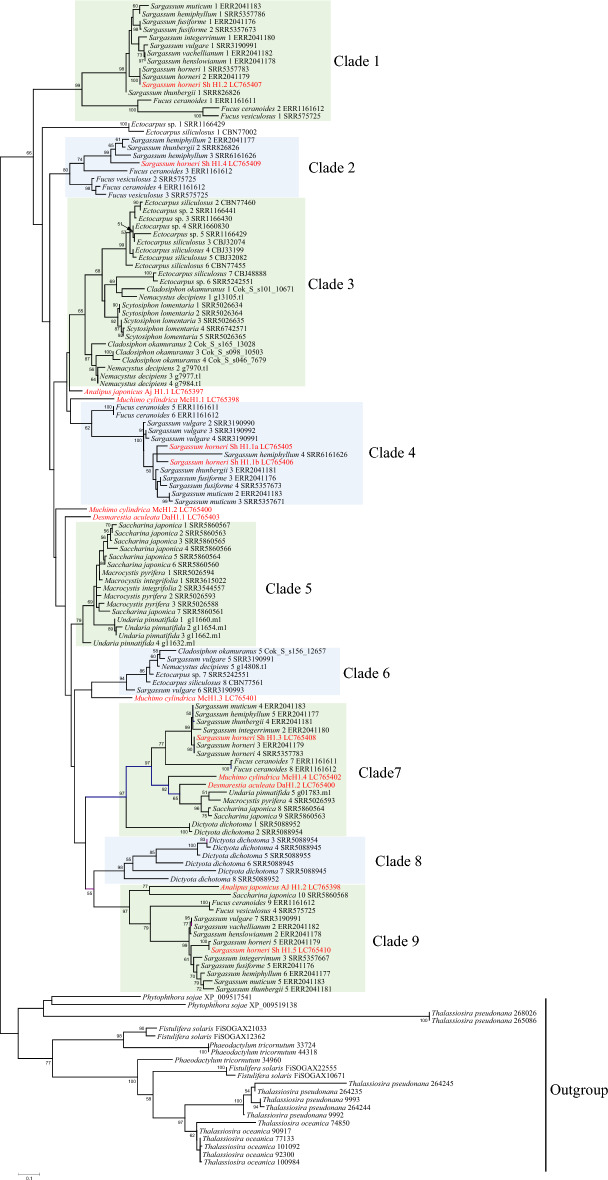
Phylogenetic tree of histone H1 of brown algae. Maximum likelihood (ML) tree inferred from histone H1 using 148 amino acid sequences. Branch support is shown above nodes as ML bootstrap values in 1000 bootstrap replicates; values below 50% were omitted. The scale bar indic-ated 0.1 changes of alignment. Grouping is based on bootstrap values and phylogenetic relationships of organisms. The dataset was constructed from transcriptomic and genomic data. The collected homologous sequences of histone H1 were aligned using mafft (alignment is shown in supplementary data) and analyzed using the maximum likelihood method in RAxML. The number after the species name indicates the histone H1 branch number of each species. The alphanumeric characters at the end of each branch represent the accession number, sequence read archive, protein ID of JGI, and gene ID (shown in Supplementary Table S4). New sequences from this study are indicated in red text.

We analyzed the phylogeny of histone H1 in brown algae to elucidate the molecular evolution of *sperm-specific Sh H1.4* using transcriptomic and genomic data on the *histone H1* gene from 7 orders of brown algae, including 13 genera and 24 species (Fig. 6). To construct a phylogenetic tree containing many lineages of brown algae, *histone H1*s were identified from *Analipus japonicas* (LC765397-8), *Mutimo cylindricus* (LC765399-402), and *Desmarestia aculeate* (LC765403-5), in addition to sequences from existing databases.

We divided histone H1s of brown algae into nine clades based on the bootstrap values and phylogenetic relationships of the organisms. All histone H1s of Fucales except for *Sargassum vulgare* were divided into clades 1, 2, 4, 7, and 9. We assigned ShH1.1a and SH1.1b to clade 4. The other four histones, ShH1.2, ShH1.3, ShH1.4, and ShH1.5, were included in clades 1, 7, 2, and 9, respectively. Three of the five clades, clades 1, 2, and 4, were composed exclusively of Fucales. Although a previous phylogenetic analysis using multiple genes suggested that Ectocarpales and Laminariales are closely related^38^, the phylogenetic tree of histone H1 showed that histone H1s of Ectocarpales and Laminariales were divided into different clades. Histone H1s of Ectocarpales were divided into clades 3 and 6, consisting only of Ectocarpales. As exceptions, the two H1 histones of *Sargassum vulgare* were included in clade 6. Among the 11 species of Fucales used in this analysis, it is difficult to suppose that only *S. vulgare* has histone H1s common to Ectocarpales. Because *Sargassum* is known to have many epiphytes attached to it^39^, we suspected that the H1s in clade 6 derived from *S. vulgare* might be due to biological contamination of an epiphytic Ectocarpales during sample collection. Comparing the diversity of histone H1 in Fucales, Ectocarpales and Laminariales, where several species were used for phylogenetic analysis, histone H1 in Fucales is divided into five clades, while in Ectocarpales and Laminariales histone H1 is divided into two clades. This result suggests that histone H1 is more diverse in Fucales than in other groups of brown algae.

## Discussion

Our results strongly suggest that the sperm nucleus of *S. horneri* contains a sperm-specific histone H1. Gametogenesis is one of the most dynamic processes of cell differentiation. In male *S. horneri*, receptacles differentiate with changing day length, and then antheridia form within them^40^. In the antheridium, nuclear condensation occurs after one nucleus has divided into 64 during spermatogenesis. However, it was not known whether brown algae sperm nuclei contain sperm-specific SNBPs, which are thought to be involved in nuclear condensation and stabilization. We revealed that *histone ShH1.4* is only expressed in the male receptacles where spermatozoa are formed. The sperm-specific expression of *ShH1.4* shown in this study should contribute to elucidating the molecular mechanisms involved in sperm nuclear condensation in brown algae and Fucales species.

An unexpected finding in the brown algal *histone H1* gene analysis was that the GD was located at the N-terminus in most of the brown algae. In general, histone H1 consists of a well-conserved GD flanked by highly basic NTD and CTD. Several studies have shown that the CTD and GDs are involved in binding to chromatin^41–44^. There are not as many reports on the function of NTDs in histone H1 as there are for CTDs, but deletion of the NTD reduces the affinity of histone H1 for DNA, suggesting that the NTD may be involved in the binding of histone H1 to DNA^45–48^. Because the gene that encodes a protein that has similarity to the CTD in bacteria, and histone H1s of protists, such as Alveolata and Euglenozoa, are composed only of the CTD, primordial histone H1 is thought to consist only of the CTD^49^. Although we cannot determine whether the brown algal H1 reported here originally lacked an NTD or lost its NTD in a common ancestor, further structural analysis of H1 including those of many Heterokontophyta species may provide new insights into the molecular evolution of H1 and the function of the NTD.

Of the three histone ShH1s comprising the SNBPs, only *ShH1.4* was not expressed in the vegetative thallus and female receptacle, suggesting it is probably a sperm nucleus-specific histone protein. The results of quantitative PCR analysis showed that the expression level of *ShH1.4* markedly increased in the receptacles containing 64-nuclei-stage antheridium. *ShH1.2* and *ShH1.3* were also significantly upregulated during spermatogenesis, but the relative increases were lower than that of *ShH1.4*. In the process of spermatogenesis of *S. horneri*, five nuclear divisions occur in the conceptacle within 2–3 days, producing 64 nuclei; therefore, the increases in expression of *ShH1.2* and *ShH1.3* may be due to the rapid progression of nuclear division in spermatogenesis. The pattern of expression changes of the *ShH1.4* gene was similar to that of the *ShMRP* gene, which encodes a protein that makes up the mastigoneme attached to the anterior flagellum of swarmer cells, including sperm of stramenopiles^36,37^. These results indicate that *ShH1.4* might be expressed in the late stage of spermatogenesis. Spatiotemporal analysis using antibodies would further elucidate the relationship between sperm-specific histone H1 and chromatin condensation in *S. horneri*.

Our previous study showed that the SDS-PAGE band pattern of histone H1s in sperm of *S. confusum* differs from that of somatic cells^29^. Therefore, the presence of sperm-specific histone H1 may be a common feature in Sargassaceae or Fucales. Phylogenetic analysis also demonstrated that Fucales had genes homologous to *ShH1.4* not found in other phylogenetic groups of brown algae.

Analysis of histone proteins in sperm nuclei and RT-PCR divided the six genes in the *S. horneri* genome detected by RNA-seq into three types. One is the sperm-specific type of *ShH1.4*. The second type consists of *ShH1.2 and ShH1.3*, which are also found in the sperm nucleus but are also expressed in somatic cells. The third type is *ShH1.1a*, *ShH1.1b*, and *ShH1.5*, which are expressed in somatic cells but not in the sperm. These results suggest that a part of the histone H1 variants protein undergoes replacement of the somatic variant for the sperm-specific histone H1 variant during spermatogenesis.

Previous reports demonstrated that sperm-specific histone H1 was involved in sperm chromatin condensation by containing more basic residues, lysine and arginine^13,50,51^. Notably, the C-terminal region of ShH1.5 has the lowest lysine composition and the highest molecular mass among the histone H1s detected in this study. In the process of spermatogenesis, the replacement of ShH1.5 with ShH1.4, which is highly basic and has a low molecular weight, may cause chromatin condensation in sperm.

ShH1.4 separated into two bands in electrophoresis (ShH1.4 and ShH1.4’), possibly because ShH1.4 undergoes post-translational modification. Post-translational modifications of histone H1s have been reported in many animals and plants^51–54^. During sea urchin spermatogenesis, dephosphorylation of sperm-specific histone H1, which was present as a phosphorylated form, was shown to lead to chromatin stabilization^18,55^. Further investigation of ShH1.4 and ShH1.4’ modifications using biochemical and molecular biological approaches may provide clues on the role of histone modifications during spermatogenesis and post-fertilization development in brown algae.

Brown algae have three types of reproductive patterns: isogamy, anisogamy, and oogamy. In contrast to the general theory of oogamy evolution^56^, isogamy is thought to be derived from oogamy in brown algae^38,57^. Regarding the morphology of the sperm nuclei, the sperm nuclei of Dictyotales and Laminariales are composed of euchromatin and heterochromatin^10,11^, whereas those of Fucales are composed only of heterochromatin^12,27^. Despite the scattering of sperm-producing brown algae species throughout the brown algae lineage^24^, the species in which significant condensation of sperm nuclei is observed are restricted to Fucales, suggesting that considerable condensation of sperm nuclei may not be an ancestral property of brown algae spermatogenesis but a property acquired by Fucales after they diverged from other brown algae. Although brown algae include several spermatogenic species, phylogenetic analysis of histone H1 also showed that sperm-specific Sh H1.4 is found in a clade composed exclusively of Fucales, suggesting that chromatin condensation in Fucales is not an ancestral trait of brown algae but a trait newly acquired in this taxon. Chromatin condensation in sperm nuclei can be considered a typical example of convergent evolution because it is observed in animals and land plants^4–8^. One of the significant features of chromatin condensation is the packing of a large genome into a compact nucleus^58,59^. Only Fucales have acquired mechanisms of dense chromatin condensation among brown algae, which may be related to their large genome size^60^. In evolving to a large genome size, an ancestor of Fucales may have evolved a sperm-specific histone H1 to create a compact sperm nucleus, thereby increasing fertilization efficiency.

In this study, we divided brown algal histone H1s into nine clades, but the evolutionary relationships between the clades were less clear. Out of the nine clades, seven were composed of histone H1s from the same order. These results may be due to the rapid evolution of histone H1^61^. Compared with other groups of brown algae, H1s of Fucales were more diverse. In the *S. horneri*, the six genes are divided into five clades, whereas the histone H1 of the *E. siliculosus* Ec32 and *Undaria pinnatifida*, whose genomes have been analyzed, are divided into three and two clades, respectively. The diversity of H1 in the Fucales may be related to the evolution of sperm-specific histone H1.

We found that only three of the six histone H1s detected by transcriptomic analysis were contained in the sperm nucleus, and that one of these three H1s, *ShH1.4*, was expressed only at the late stage of spermatogenesis. Although sperm-specific histone H1 has been reported in animals, to the best of our knowledge this is the first report of its existence in a lineage group other than animals. In the future, detailed analysis of the expression timing using antibodies and DNA affinity should help to elucidate the relationship between ShH1.4 and the chromatin condensation mechanism in the sperm nucleus of *S. horneri.*

## Materials and methods

### Materials

Male and female matured thalli of *Sargassum horneri* for cultivation, SNBP analysis, and total RNA extraction for reverse transcription and quantitative PCR were collected in April and May from 2018 to 2020 at Shikimi-beach, Sekumi, Wakasa-cho, Mikatakaminaka-gun, Fukui, Japan (35°N 135°E). After collection, the samples were brought back to the laboratory within an hour to check the progress of spermatogenesis. The receptacles were removed from the thallus and washed with filtrated seawater more than three times. The progress of spermatogenesis in male receptacles (1- or 64-nuclei stage) and oogenesis in female receptacles was judged by microscopic observation of antheridium and oogonium, respectively. The receptacles were frozen with liquid nitrogen and stored at − 80 °C before total RNA extraction from the male and female receptacles. Male receptacles containing 64-nuclei-stage antheridium were used for sperm liberation. The procedure of sperm liberation was performed as described previously^62^. The culture strain of *S. horneri* was established from the zygote on the surface of the receptacle collected on April 30, 2018.

*Analipus japonicus* (KU-0883), *Mutimo cylindricus* (KU-0761), and *Desmarestia aculeate* (KU-1140) were obtained from The Kobe University Macro-Algal Culture Collection (KU-MACC).

### Culture conditions

The culture conditions for vegetative growth of *S. horneri* were as described in a previous report^62^. Cultivated thallus was used for total RNA extraction of the vegetative thallus. *A. japonicus*, *M. cylindricus*, and *D. aculeate* were cultured in modified Provasoli Enriched Seawater (PES) medium with constant aeration (approximately 800 mL min^−1^) at 15 °C and 16 h light (40 μmol m^−2^ s^−1^, daylight-type fluorescent lamps) and 8 h dark cycles.

### Light and fluorescent microscopy observations

The preparation of samples for observations of antheridium by light and fluorescent microscopy was performed as described previously^27^. Each specimen was stained with DAPI (0.25 μg/ml) and mounted in ibidi mounting medium (ibidi, Germany). Microphotographs were taken using a BX 51 microscope (Olympus, Tokyo, Japan) equipped with a DP70 digital camera (Olympus).

### Identification of histone H1 in *Sargassum horneri*

We identified histone H1 of *S. horneri* by performing a blast search against the transcript database of *S. horneri* (DDBJ accession no. PRJDB4109) with histone H1 of *E. siliculosus* as a query.

### Isolation of nuclei of sperm of* S. horneri*

Swimming sperm of *S. horneri* were filtered through Miracloth (475,855-1R; Merck, Boston, MA, USA) and centrifuged at 4 °C and 7000 rpm for 10 min. The cell pellet was suspended in a nuclear isolation buffer as described previously^28^ and homogenized with a Potter–Elvehjem grinder. The suspension was centrifuged at 4 °C and 10,000 rpm for 10 min. The pellet was resuspended with nuclear isolation buffer and homogenized. After two rounds of centrifugation and homogenization, the crude nuclear fraction was obtained.

### Extraction of basic proteins

The basic proteins were extracted with 0.2 M H_2_SO_4_ overnight at 4 °C. After centrifugation (14,000 rpm, 15 min), the acid-soluble proteins were precipitated by the addition of 100% trichloroacetic acid, giving a final concentration of 20% in the supernatant, and chilled for 1 h on ice. The precipitates were collected by centrifugation (14,000 rpm, 15 min, 4 °C) and the pellet was washed twice with acetone and stored at − 80℃.

### Electrophoresis

SDS-PAGE and two-dimensional electrophoresis were performed as in a previous study^28^. Sperm basic proteins extracted from sperm nuclei of *S. horneri* were separated by SDS-PAGE (14% polyacrylamide gel). Gels were stained using one-step CBB staining solution (Biocraft, Tokyo, Japan).

### In-gel digestion and LC–MS/MS analysis

In-gel digestion and LC–MS/MS analysis were performed in accordance with the work of Fu et al.^63^. Sperm basic proteins extracted from sperm nuclei of *S. horneri* were separated by SDS-PAGE (14% polyacrylamide gel). Gels were stained using CBB staining solution (SP-4010; Integrale, Tokushima, Japan). Four bands around 25 kDa (Fig. 4) were cut using a razor blade. The excised bands were digested with trypsin. The tryptic digests were separated by Paradigm MS2 HPLC (Bruker-Michrom, Auburn, CA, USA) equipped with an HTS-PAL auto-sample injection system (LEAP, Carrboro, NC, USA) on a nanocapillary column (0.1 mm inner diameter × 50 mm; Chemicals Evaluation and Research Institute, Tokyo, Japan). The eluates from the column were subsequently subjected to mass spectral analysis using an Orbitrap XL mass spectrometer (Thermo Scientific, Waltham, MA, USA). MS/MS spectral data were analyzed using Thermo Proteome Discoverer version 1.4.0.208 with the Mascot search engine (Matrix Science, London, UK), using transcriptomic data of *S. horneri*^35^.

### Total RNA extraction

The procedure for total RNA extraction was performed as described previously^35^. RNA extracted from *A. japonicus*, *M. cylindricus*, and *D. aculeate* was subjected to RNA-seq analysis. In the extraction of RNA from individuals with the receptacles of *S. horneri*, the condition of the spermatogonia nuclei was checked under a fluorescence microscope and receptacles containing conceptacles with condensed nuclei were defined as 64-nuclei-stage receptacles.

### RNA sequencing and assembly

The RNA-Seq of all of the samples, from *A. japonicus*, *M. cylindricus*, and *D. aculeata*, was outsourced to Eurofins Genomics (Tokyo, Japan), using an Illumina TruSeq RNA sample preparation kit (Illumina, Inc., San Diego, CA, USA) for library preparation, and an Illumina NovaSeq 6000 for the transcriptome sequence (150 bp, paired-end). Along with the RNA-Seq data newly generated as described above, we also obtained raw reads of brown algae from the Sequence Read Archive in NCBI (https://www.ncbi.nlm.nih.gov/sra, accessed December 2019, see Fig. 6, SupplymentalyTable S3) using fasterq-dump.2.9.6^64^. The sequence reads in each dataset were trimmed using fastp v0.20.0^65^ (with parameters: -q 30, -n 10, -t 1, -T 1, -l 20, -w 16) and assembled with Trinity 2.4.0^66^. The assembled transcripts were clustered with CDhit v4.8.1^67^ to remove redundancy (parameters: -c 0.95, -T 8, -M 8000).

### Phylogenetic analysis

To reconstruct the phylogeny of histone H1 in brown algae, we retrieved the histone H1 sequences from the transcriptome assemblies, using that of the fully curated genome of *E. siliculosus* Ec 32 as a query for a Local Blast search with an e-value threshold of 1e − 30. All of the collected DNA sequences were then translated into amino acid sequences with Transdecoder (v5.5.0 using the default parameters)^68^. We also obtained amino acid sequences of histone H1 from publicly available, well-annotated whole-genome sequences, including 2 brown algae (*Cladosiphon okamuranus*^69^ and *Nemacystus decipiens*^70^) as well as 4 diatoms (*Thalassiosira pseudonana*, *T. oceanica*, *Phaeodactylum tricornutum*, and *Fistulifera solaris*) and a non-photosynthetic stramenopile (*Phytophthora sojae*) as an outgroup. Furthermore, the amino acid sequence of histone H1 of *U. pinnatifida* was kindly provided by Dr. Tifeng Shan^71^.

All of the amino acid sequences of histone H1 from brown algae and outgroups were aligned using Mafft v7.455 (shown as supplementary alignment data)^72^. Then, we filtered sequences to remove redundancy and only leave representative sequences based on the following criteria: (1) starting with methionine; (2) > 100 and < 300 amino acids in length; (3) the longest one among isoforms of a given gene annotated by Trinity; and (4) the alignment was trimmed using TrimAL v1.4.1^73^ (parameter: -gt 0.9). We further removed sequences to leave one representative in cases in which multiple sequences from a genome/transcriptome were identical at this stage. RAxML 8.2.10^74^ was used for maximum likelihood analyses with the PROTGAMMAWAG model, for which gamma correction values were obtained automatically by the program. The best scoring ML tree was obtained with 200 replicates of hill-climbing searches; we performed 1,000 bootstrap analyses. The phylogenetic tree was edited by MEGA7^75^.

### Gene expression analysis (qRT-PCR, RT-PCR)

The synthesis of complementary DNA (cDNA) for qRT-PCR and RT-PCR was performed using the PrimeScript RT Reagent Kit (Takara, Kusatsu, Japan). Quantitative RT-PCR was performed using FastStart SYBRGreen Master (Roche Diagnostics K.K., Tokyo, Japan). Data were analyzed by Light Cycler 96 System (Roche Diagnostics) and actin gene expression was used as an internal control. The cDNA was amplified by RT-PCR for 36 cycles. The primer sequences and predicted amplification product size are shown in Supplementary Table S2. Relative expression levels were calculated using The E-Method^76^.

### Data analysis

Gene expression data were analyzed by one-way ANOVA, followed by Tukey’s test, using JSTAT v.13.0.

### Supplementary Information

Supplementary Information.

Supplementary Figures.

Supplementary Information.

Supplementary Figures.

Supplementary Table S1.

Supplementary Table S2.

Supplementary Table S3.

Supplementary Table S4.

## Data Availability

The *histone H1* sequences presented in this paper have been deposited in the DDBJ (LC765397, LC765398, LC765399, LC765400, LC765401, LC765402, LC765403, LC765404, LC765405, LC765406, LC765407, LC765408, LC765409, LC765410) as nucleotide sequences https://www.ncbi.nlm.nih.gov/nuccore/LC765397,LC765398,LC765399,LC765400,LC765401,LC765402,LC765403,LC765404,LC765405,LC765406,LC765407,LC765408,LC765409,LC765410. To obtain the data used in this paper, please contact the corresponding author.
