# Supplementary material for: Sperm-specific histone H1 in highly condensed sperm nucleus of Sargassum horneri
Source: Sci Rep. 2024 Feb 9;14:3387. doi: 10.1038/s41598-024-53729-2 (PMC10858212; doi:10.1038/s41598-024-53729-2)
Supplement: Supplementary file 2 — Supplementary Figures. [file 41598_2024_53729_MOESM2_ESM.pptx]

## Slide 1
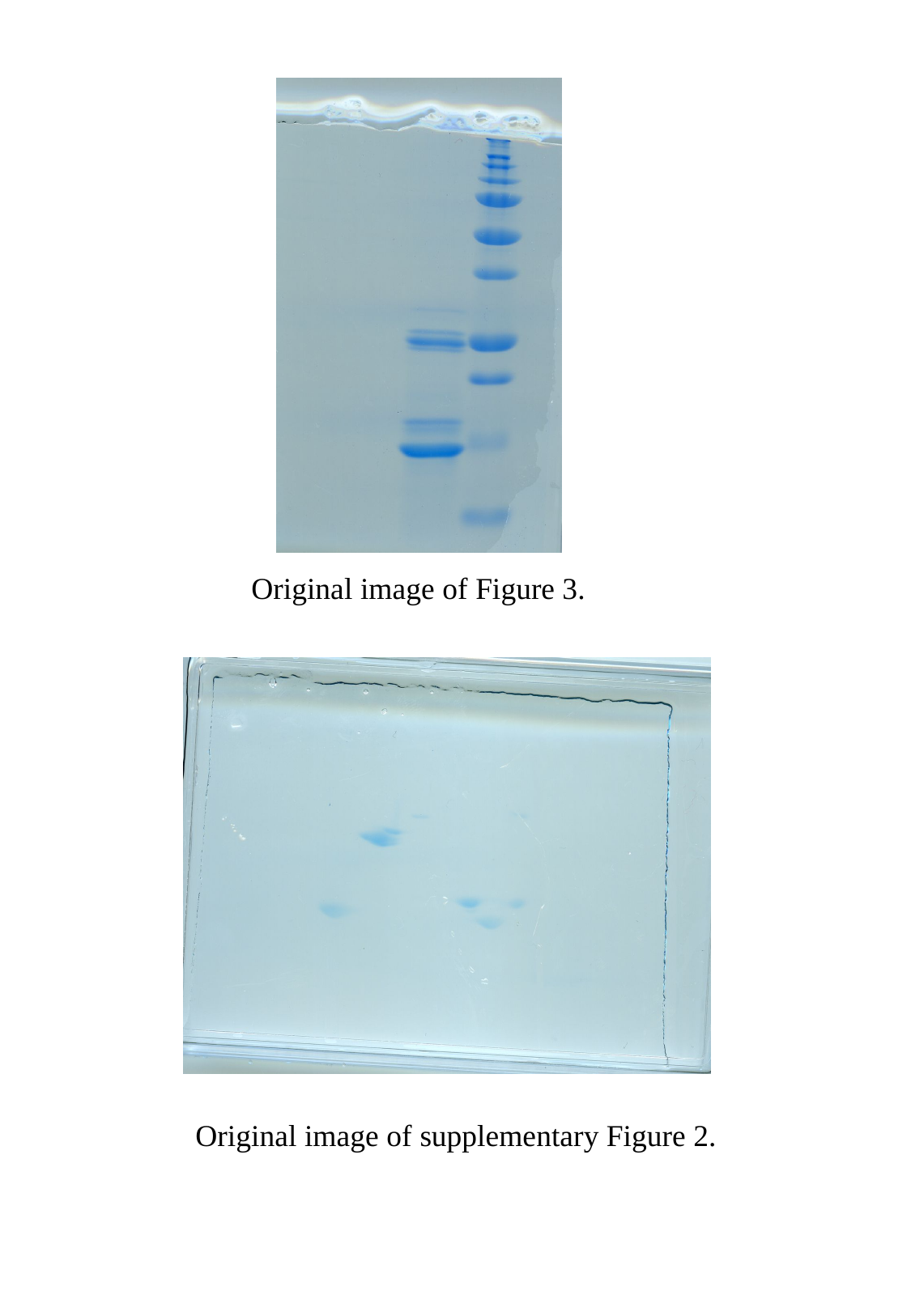

Original image of Figure 3.
Original image of supplementary Figure 2.

## Slide 2
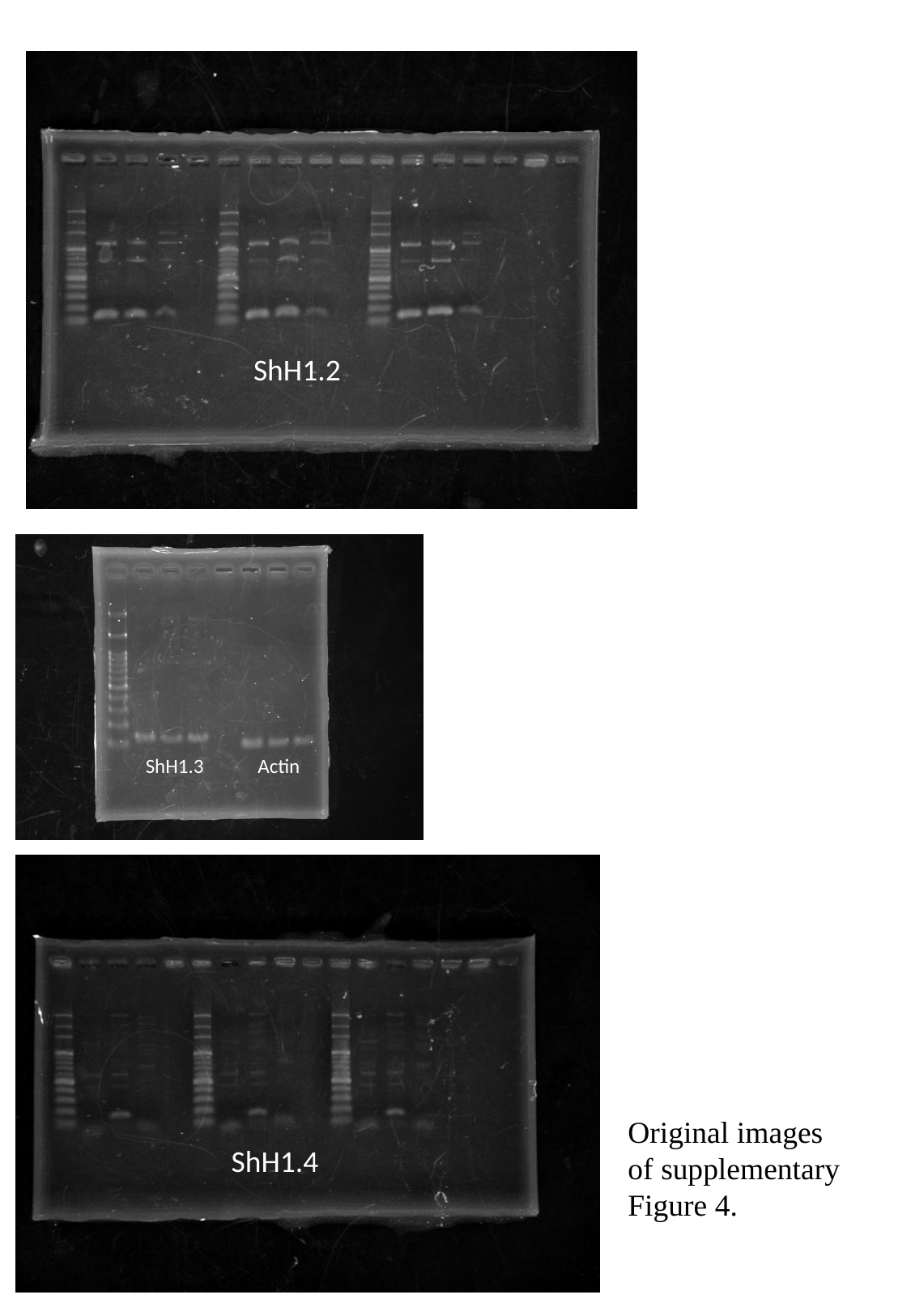

ShH1.2
ShH1.3
Actin
Original images of supplementary Figure 4.
ShH1.4

## Slide 3
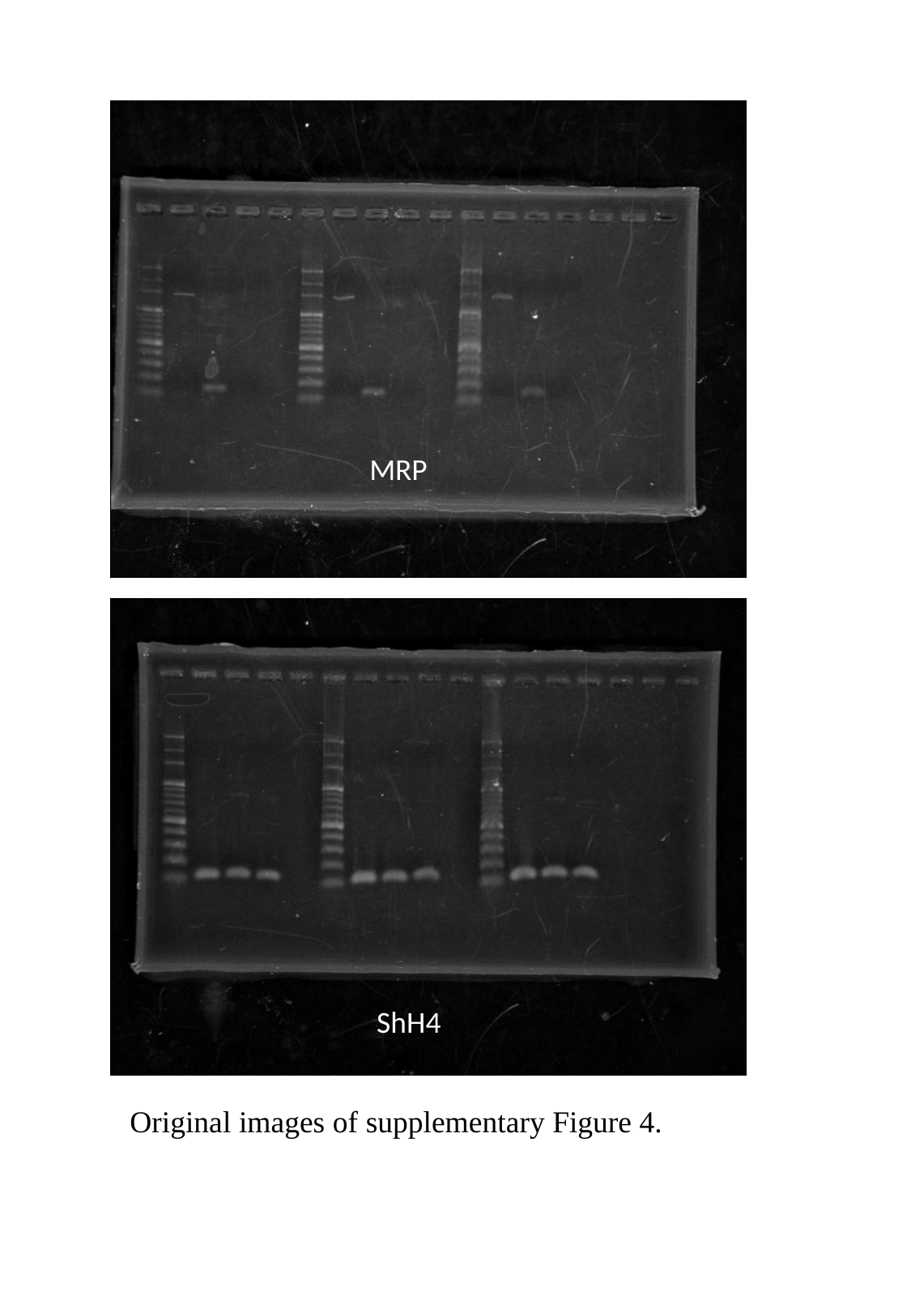

MRP
ShH4
Original images of supplementary Figure 4.

## Slide 4
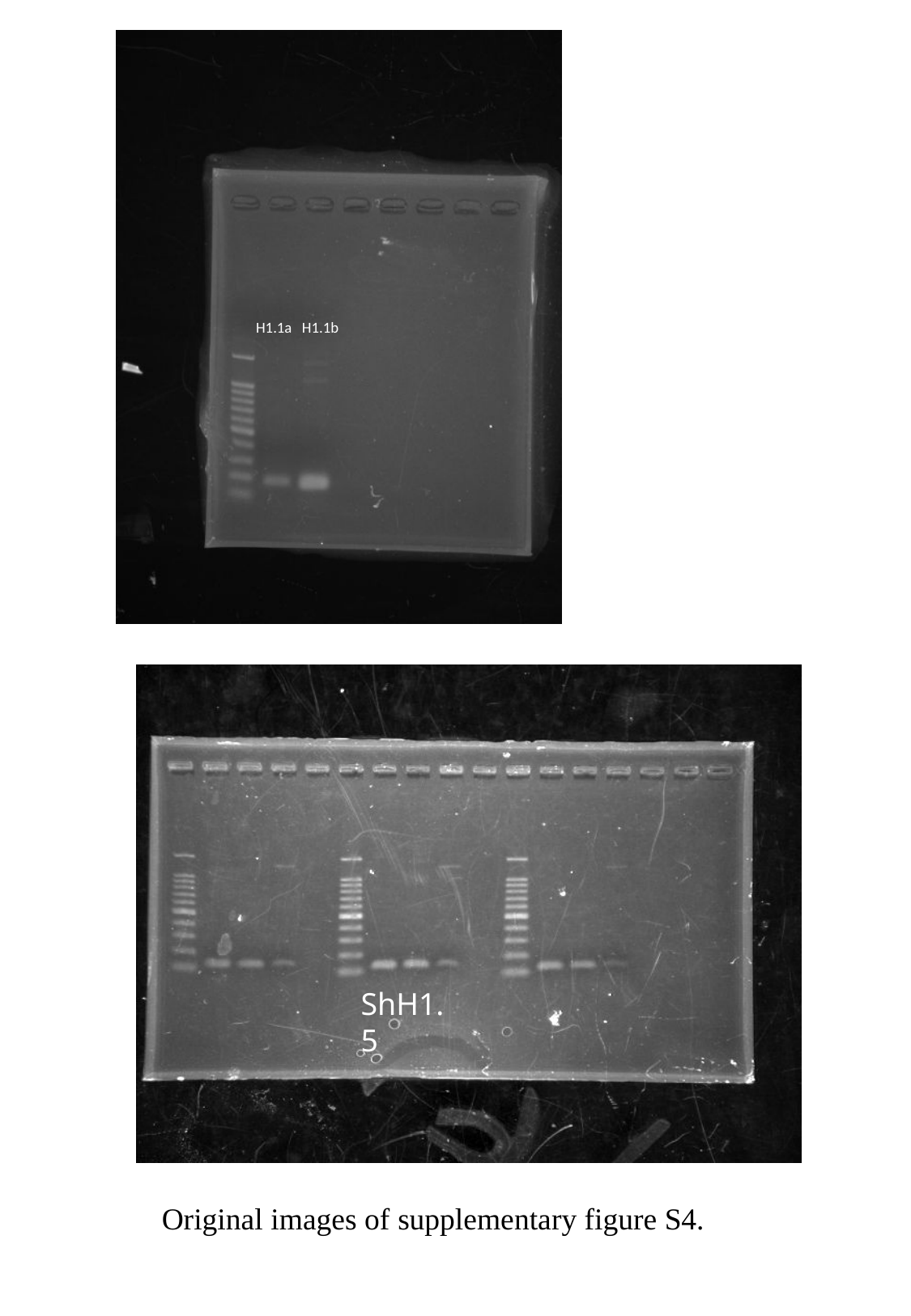

H1.1a
H1.1b
ShH1.5
Original images of supplementary figure S4.
