# Supplementary material for: Sperm-specific histone H1 in highly condensed sperm nucleus of Sargassum horneri
Source: Sci Rep. 2024 Feb 9;14:3387. doi: 10.1038/s41598-024-53729-2 (PMC10858212; doi:10.1038/s41598-024-53729-2)
Supplement: Supplementary file 4 — Supplementary Figures. [file 41598_2024_53729_MOESM4_ESM.pptx]

## Slide 1
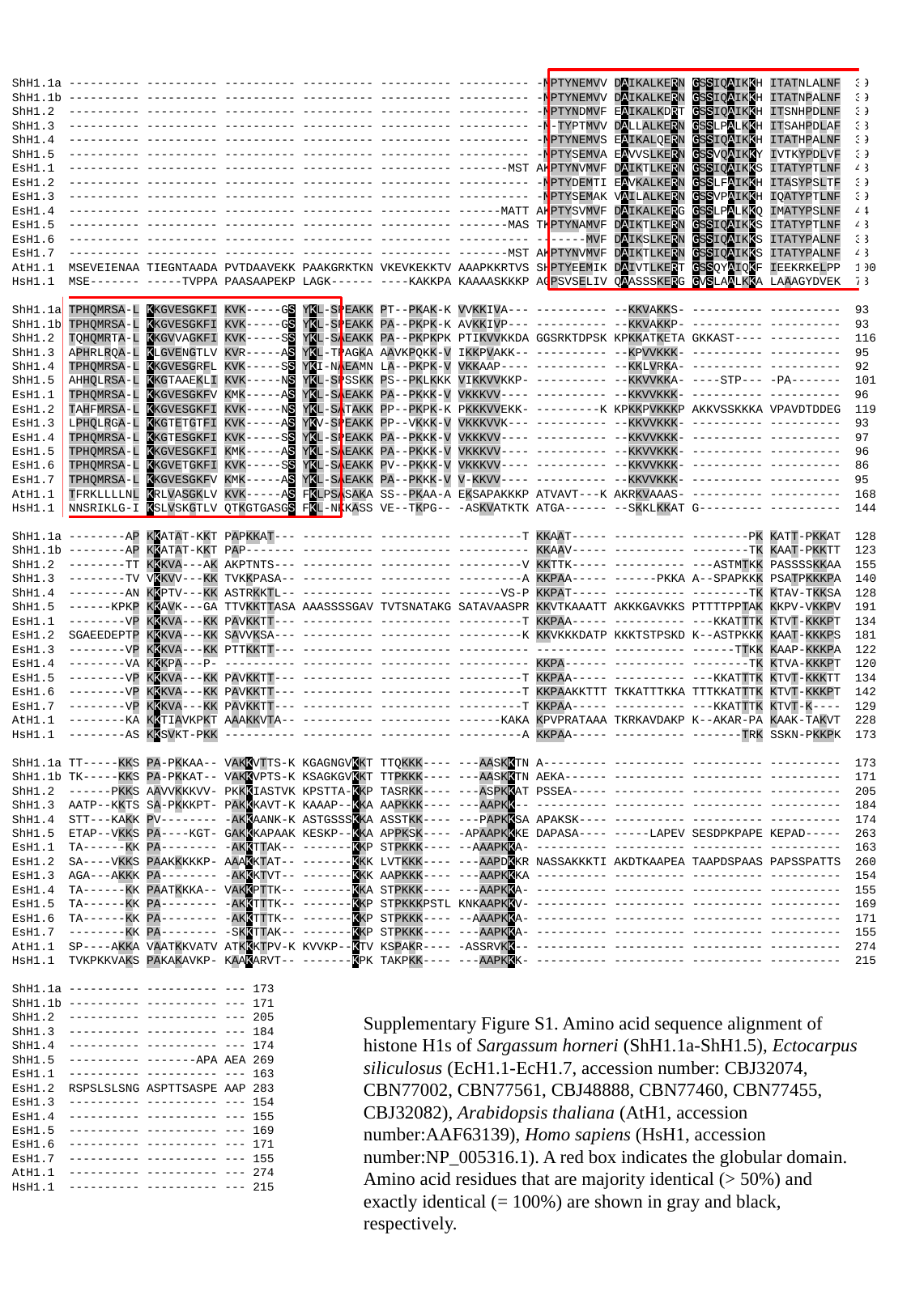

ShH1.1a ---------- ---------- ---------- ---------- ---------- ---------- -MPTYNEMVV DAIKALKERN GSSIQAIKKH ITATNLALNF 39
ShH1.1b ---------- ---------- ---------- ---------- ---------- ---------- -MPTYNEMVV DAIKALKERN GSSIQAIKKH ITATNPALNF 39
ShH1.2 ---------- ---------- ---------- ---------- ---------- ---------- -MPTYNDMVF EAIKALKDRT GSSIQAIKKH ITSNHPDLNF 39
ShH1.3 ---------- ---------- ---------- ---------- ---------- ---------- -M-TYPTMVV DALLALKERN GSSLPALKKH ITSAHPDLAF 38
ShH1.4 ---------- ---------- ---------- ---------- ---------- ---------- -MPTYNEMVS EAIKALQERN GSSIQAIKKH ITATHPALNF 39
ShH1.5 ---------- ---------- ---------- ---------- ---------- ---------- -MPTYSEMVA EAVVSLKERN GSSVQAIKKY IVTKYPDLVF 39
EsH1.1 ---------- ---------- ---------- ---------- ---------- -------MST AKPTYNVMVF DAIKTLKERN GSSIQAIKKS ITATYPTLNF 43
EsH1.2 ---------- ---------- ---------- ---------- ---------- ---------- -MPTYDEMTI EAVKALKERN GSSLFAIKKH ITASYPSLTF 39
EsH1.3 ---------- ---------- ---------- ---------- ---------- ---------- -MPTYSEMAK VAILALKERN GSSVPAIKKH IQATYPTLNF 39
EsH1.4 ---------- ---------- ---------- ---------- ---------- ------MATT AKPTYSVMVF DAIKALKERG GSSLPALKKQ IMATYPSLNF 44
EsH1.5 ---------- ---------- ---------- ---------- ---------- -------MAS TKPTYNAMVF DAIKTLKERN GSSIQAIKKS ITATYPTLNF 43
EsH1.6 ---------- ---------- ---------- ---------- ---------- ---------- -------MVF DAIKSLKERN GSSIQAIKKS ITATYPALNF 33
EsH1.7 ---------- ---------- ---------- ---------- ---------- -------MST AKPTYNVMVF DAIKTLKERN GSSIQAIKKS ITATYPALNF 43
AtH1.1 MSEVEIENAA TIEGNTAADA PVTDAAVEKK PAAKGRKTKN VKEVKEKKTV AAAPKKRTVS SHPTYEEMIK DAIVTLKERT GSSQYAIQKF IEEKRKELPP 100
HsH1.1 MSE------- -----TVPPA PAASAAPEKP LAGK------ ----KAKKPA KAAAASKKKP AGPSVSELIV QAASSSKERG GVSLAALKKA LAAAGYDVEK 78
ShH1.1a TPHQMRSA-L KKGVESGKFI KVK-----GS YKL-SPEAKK PT--PKAK-K VVKKIVA--- ---------- --KKVAKKS- ---------- ---------- 93
ShH1.1b TPHQMRSA-L KKGVESGKFI KVK-----GS YKL-SPEAKK PA--PKPK-K AVKKIVP--- ---------- --KKVAKKP- ---------- ---------- 93
ShH1.2 TQHQMRTA-L KKGVVAGKFI KVK-----SS YKL-SAEAKK PA--PKPKPK PTIKVVKKDA GGSRKTDPSK KPKKATKETA GKKAST---- ---------- 116
ShH1.3 APHRLRQA-L KLGVENGTLV KVR-----AS YKL-TPAGKA AAVKPQKK-V IKKPVAKK-- ---------- --KPVVKKK- ---------- ---------- 95
ShH1.4 TPHQMRSA-L KKGVESGRFL KVK-----SS YKI-NAEAMN LA--PKPK-V VKKAAP---- ---------- --KKLVRKA- ---------- ---------- 92
ShH1.5 AHHQLRSA-L KKGTAAEKLI KVK-----NS YKL-SPSSKK PS--PKLKKK VIKKVVKKP- ---------- --KKVVKKA- ----STP--- -PA------- 101
EsH1.1 TPHQMRSA-L KKGVESGKFV KMK-----AS YKL-SAEAKK PA--PKKK-V VKKKVV---- ---------- --KKVVKKK- ---------- ---------- 96
EsH1.2 TAHFMRSA-L KKGVESGKFI KVK-----NS YKL-SATAKK PP--PKPK-K PKKKVVEKK- ---------K KPKKPVKKKP AKKVSSKKKA VPAVDTDDEG 119
EsH1.3 LPHQLRGA-L KKGTETGTFI KVK-----AS YKV-SPEAKK PP--VKKK-V VKKKVVK--- ---------- --KKVVKKK- ---------- ---------- 93
EsH1.4 TPHQMRSA-L KKGTESGKFI KVK-----SS YKL-SPEAKK PA--PKKK-V VKKKVV---- ---------- --KKVVKKK- ---------- ---------- 97
EsH1.5 TPHQMRSA-L KKGVESGKFI KMK-----AS YKL-SAEAKK PA--PKKK-V VKKKVV---- ---------- --KKVVKKK- ---------- ---------- 96
EsH1.6 TPHQMRSA-L KKGVETGKFI KVK-----SS YKL-SAEAKK PV--PKKK-V VKKKVV---- ---------- --KKVVKKK- ---------- ---------- 86
EsH1.7 TPHQMRSA-L KKGVESGKFV KMK-----AS YKL-SAEAKK PA--PKKK-V V-KKVV---- ---------- --KKVVKKK- ---------- ---------- 95
AtH1.1 TFRKLLLLNL KRLVASGKLV KVK-----AS FKLPSASAKA SS--PKAA-A EKSAPAKKKP ATVAVT---K AKRKVAAAS- ---------- ---------- 168
HsH1.1 NNSRIKLG-I KSLVSKGTLV QTKGTGASGS FKL-NKKASS VE--TKPG-- -ASKVATKTK ATGA------ --SKKLKKAT G--------- ---------- 144
ShH1.1a --------AP KKATAT-KKT PAPKKAT--- ---------- ---------- ---------T KKAAT----- ---------- --------PK KATT-PKKAT 128
ShH1.1b --------AP KKATAT-KKT PAP------- ---------- ---------- ---------- KKAAV----- ---------- --------TK KAAT-PKKTT 123
ShH1.2 --------TT KKKVA---AK AKPTNTS--- ---------- ---------- ---------V KKTTK----- ---------- ---ASTMTKK PASSSSKKAA 155
ShH1.3 --------TV VKKVV---KK TVKKPASA-- ---------- ---------- ---------A KKPAA----- ------PKKA A--SPAPKKK PSATPKKKPA 140
ShH1.4 --------AN KKPTV---KK ASTRKKTL-- ---------- ---------- ------VS-P KKPAT----- ---------- --------TK KTAV-TKKSA 128
ShH1.5 ------KPKP KKAVK---GA TTVKKTTASA AAASSSSGAV TVTSNATAKG SATAVAASPR KKVTKAAATT AKKKGAVKKS PTTTTPPTAK KKPV-VKKPV 191
EsH1.1 --------VP KKKVA---KK PAVKKTT--- ---------- ---------- ---------T KKPAA----- ---------- ---KKATTTK KTVT-KKKPT 134
EsH1.2 SGAEEDEPTP KKKVA---KK SAVVKSA--- ---------- ---------- ---------K KKVKKKDATP KKKTSTPSKD K--ASTPKKK KAAT-KKKPS 181
EsH1.3 --------VP KKKVA---KK PTTKKTT--- ---------- ---------- ---------- ---------- ---------- ------TTKK KAAP-KKKPA 122
EsH1.4 --------VA KKKPA---P- ---------- ---------- ---------- ---------- KKPA------ ---------- --------TK KTVA-KKKPT 120
EsH1.5 --------VP KKKVA---KK PAVKKTT--- ---------- ---------- ---------T KKPAA----- ---------- ---KKATTTK KTVT-KKKTT 134
EsH1.6 --------VP KKKVA---KK PAVKKTT--- ---------- ---------- ---------T KKPAAKKTTT TKKATTTKKA TTTKKATTTK KTVT-KKKPT 142
EsH1.7 --------VP KKKVA---KK PAVKKTT--- ---------- ---------- ---------T KKPAA----- ---------- ---KKATTTK KTVT-K---- 129
AtH1.1 --------KA KKTIAVKPKT AAAKKVTA-- ---------- ---------- ------KAKA KPVPRATAAA TKRKAVDAKP K--AKAR-PA KAAK-TAKVT 228
HsH1.1 --------AS KKSVKT-PKK ---------- ---------- ---------- ---------A KKPAA----- ---------- -------TRK SSKN-PKKPK 173
ShH1.1a TT-----KKS PA-PKKAA-- VAKKVTTS-K KGAGNGVKKT TTQKKK---- ---AASKKTN A--------- ---------- ---------- ---------- 173
ShH1.1b TK-----KKS PA-PKKAT-- VAKKVPTS-K KSAGKGVKKT TTPKKK---- ---AASKKTN AEKA------ ---------- ---------- ---------- 171
ShH1.2 ------PKKS AAVVKKKVV- PKKKIASTVK KPSTTA-KKP TASRKK---- ---ASPKKAT PSSEA----- ---------- ---------- ---------- 205
ShH1.3 AATP--KKTS SA-PKKKPT- PAKKKAVT-K KAAAP--KKA AAPKKK---- ---AAPKK-- ---------- ---------- ---------- ---------- 184
ShH1.4 STT---KAKK PV-------- -AKKAANK-K ASTGSSSKKA ASSTKK---- ---PAPKKSA APAKSK---- ---------- ---------- ---------- 174
ShH1.5 ETAP--VKKS PA----KGT- GAKKKAPAAK KESKP--KKA APPKSK---- -APAAPKKKE DAPASA---- -----LAPEV SESDPKPAPE KEPAD----- 263
EsH1.1 TA------KK PA-------- -AKKTTAK-- -------KKP STPKKK---- --AAAPKKA- ---------- ---------- ---------- ---------- 163
EsH1.2 SA----VKKS PAAKKKKKP- AAAKKTAT-- -------KKK LVTKKK---- ---AAPDKKR NASSAKKKTI AKDTKAAPEA TAAPDSPAAS PAPSSPATTS 260
EsH1.3 AGA---AKKK PA-------- -AKKKTVT-- -------KKK AAPKKK---- ---AAPKKKA ---------- ---------- ---------- ---------- 154
EsH1.4 TA------KK PAATKKKA-- VAKKPTTK-- -------KKA STPKKK---- ---AAPKKA- ---------- ---------- ---------- ---------- 155
EsH1.5 TA------KK PA-------- -AKKTTTK-- -------KKP STPKKKPSTL KNKAAPKKV- ---------- ---------- ---------- ---------- 169
EsH1.6 TA------KK PA-------- -AKKTTTK-- -------KKP STPKKK---- --AAAPKKA- ---------- ---------- ---------- ---------- 171
EsH1.7 --------KK PA-------- -SKKTTAK-- -------KKP STPKKK---- ---AAPKKA- ---------- ---------- ---------- ---------- 155
AtH1.1 SP----AKKA VAATKKVATV ATKKKTPV-K KVVKP--KTV KSPAKR---- -ASSRVKK-- ---------- ---------- ---------- ---------- 274
HsH1.1 TVKPKKVAKS PAKAKAVKP- KAAKARVT-- -------KPK TAKPKK---- ---AAPKKK- ---------- ---------- ---------- ---------- 215
ShH1.1a ---------- ---------- --- 173
ShH1.1b ---------- ---------- --- 171
ShH1.2 ---------- ---------- --- 205
ShH1.3 ---------- ---------- --- 184
ShH1.4 ---------- ---------- --- 174
ShH1.5 ---------- -------APA AEA 269
EsH1.1 ---------- ---------- --- 163
EsH1.2 RSPSLSLSNG ASPTTSASPE AAP 283
EsH1.3 ---------- ---------- --- 154
EsH1.4 ---------- ---------- --- 155
EsH1.5 ---------- ---------- --- 169
EsH1.6 ---------- ---------- --- 171
EsH1.7 ---------- ---------- --- 155
AtH1.1 ---------- ---------- --- 274
HsH1.1 ---------- ---------- --- 215
Supplementary Figure S1. Amino acid sequence alignment of histone H1s of Sargassum horneri (ShH1.1a-ShH1.5), Ectocarpus siliculosus (EcH1.1-EcH1.7, accession number: CBJ32074, CBN77002, CBN77561, CBJ48888, CBN77460, CBN77455, CBJ32082), Arabidopsis thaliana (AtH1, accession number:AAF63139), Homo sapiens (HsH1, accession number:NP_005316.1). A red box indicates the globular domain. Amino acid residues that are majority identical (> 50%) and exactly identical (= 100%) are shown in gray and black, respectively.

## Slide 2
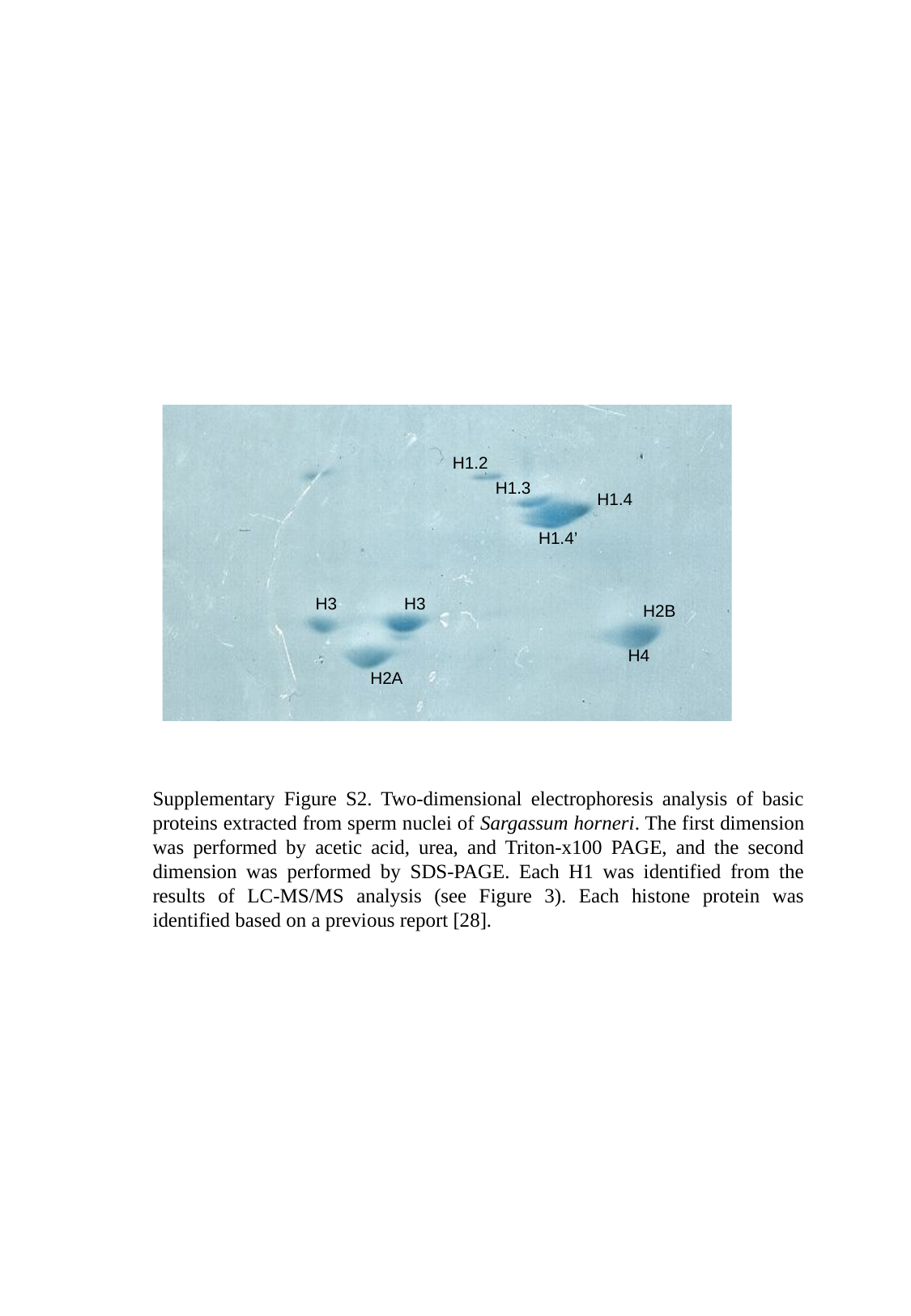

H1.2
H1.3
H1.4
H1.4’
H3
H3
H2B
H4
H2A
Supplementary Figure S2. Two-dimensional electrophoresis analysis of basic proteins extracted from sperm nuclei of Sargassum horneri. The first dimension was performed by acetic acid, urea, and Triton-x100 PAGE, and the second dimension was performed by SDS-PAGE. Each H1 was identified from the results of LC-MS/MS analysis (see Figure 3). Each histone protein was identified based on a previous report [28].

## Slide 3
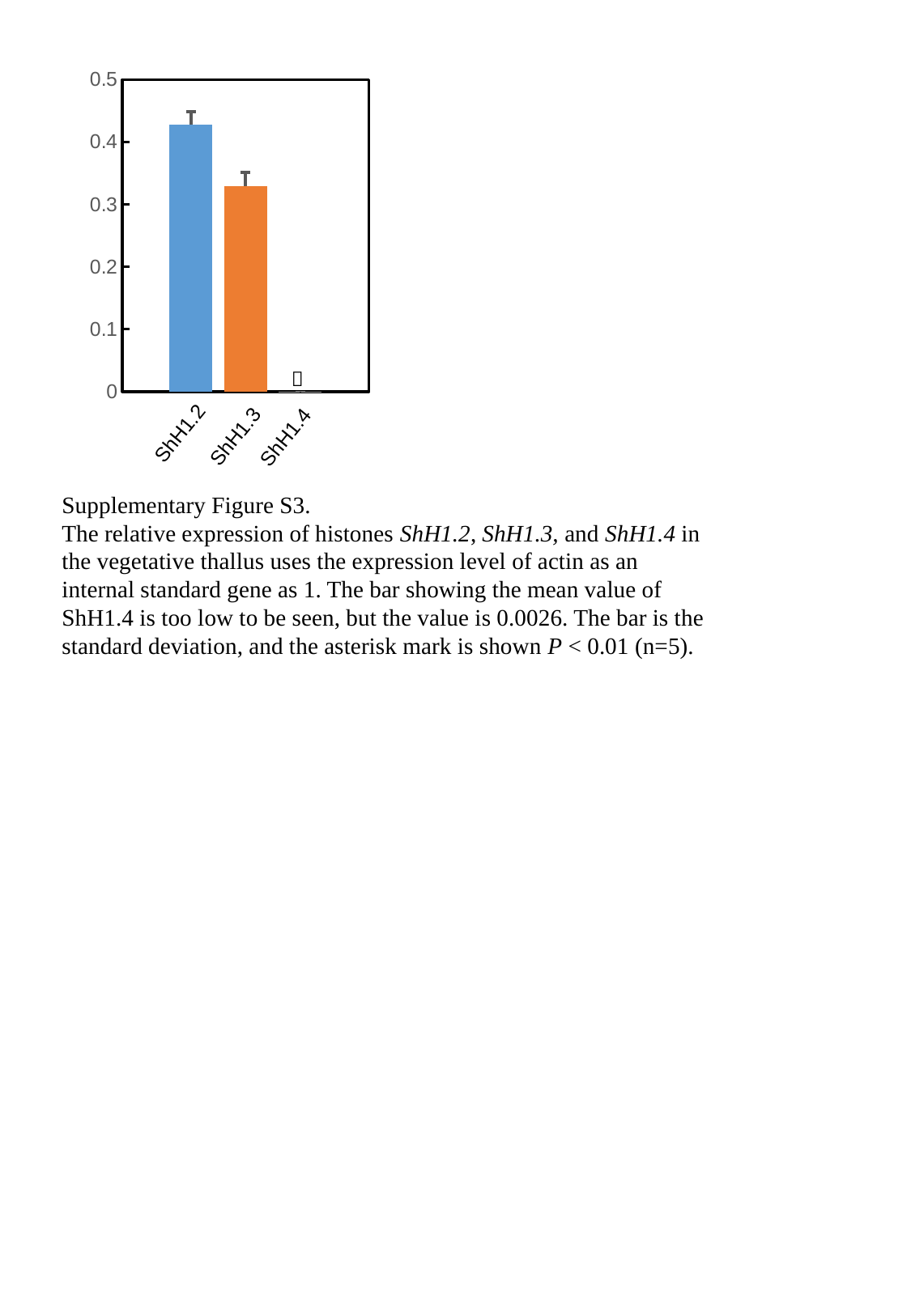

### Chart
| Category | ShH1.2 | ShH1.3 | ShH1.4 |
|---|---|---|---|＊
ShH1.2
ShH1.3
ShH1.4
Supplementary Figure S3.
The relative expression of histones ShH1.2, ShH1.3, and ShH1.4 in the vegetative thallus uses the expression level of actin as an internal standard gene as 1. The bar showing the mean value of ShH1.4 is too low to be seen, but the value is 0.0026. The bar is the standard deviation, and the asterisk mark is shown P < 0.01 (n=5).

## Slide 4
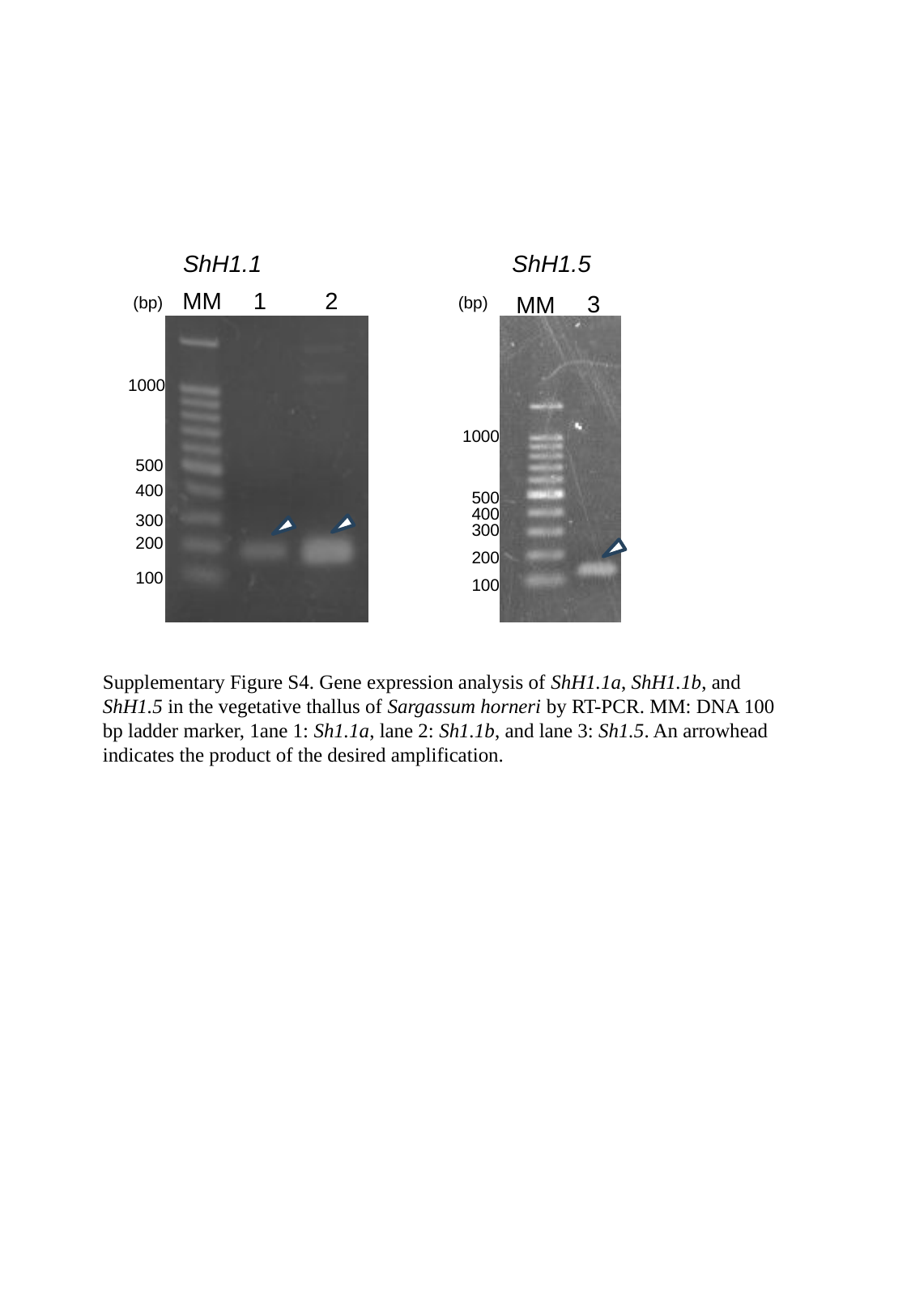

ShH1.1
ShH1.5
MM
1
2
3
MM
(bp)
(bp)
1000
1000
500
400
500
400
300
300
200
200
100
100
Supplementary Figure S4. Gene expression analysis of ShH1.1a, ShH1.1b, and ShH1.5 in the vegetative thallus of Sargassum horneri by RT-PCR. MM: DNA 100 bp ladder marker, 1ane 1: Sh1.1a, lane 2: Sh1.1b, and lane 3: Sh1.5. An arrowhead indicates the product of the desired amplification.
