# Supplementary material for: Sperm-specific histone H1 in highly condensed sperm nucleus of Sargassum horneri
Source: Sci Rep. 2024 Feb 9;14:3387. doi: 10.1038/s41598-024-53729-2 (PMC10858212; doi:10.1038/s41598-024-53729-2)
Supplement: Supplementary file 5 — Supplementary Table S1. [file 41598_2024_53729_MOESM5_ESM.pdf]

Supplementary Table S1. Sequence identity of histone H1s.

Sequence identity (%) of globular domain

	ShH1.1a	ShH1.1b	ShH1.2	ShH1.3	ShH1.4	ShH1.5	EcH1.1	AtH1.1	HsH1.1
ShH1.1a		98.5	77.6	61.2	85.1	64.2	83.6	49.3	29.2
ShH1.1b			79.1	62.7	86.6	65.7	85.1	49.3	29.2
ShH1.2				58.2	76.1	64.2	73.1	44.9	23.6
ShH1.3					58.2	55.2	58.2	42.0	33.3
ShH1.4						59.7	77.6	42.0	25.0
ShH1.5							61.2	47.8	27.8
EcH1.1								50.7	26.4
AtH1.1									31.1
HsH1.1									

Sequence identity (%) of C-terminal domain

	ShH1.1a	ShH1.1b	ShH1.2	ShH1.3	ShH1.4	ShH1.5	EcH1.1	AtH1.1	HsH1.1
ShH1.1a		81.1	39.8	37.4	40.0	29.8	49.1	27.3	21.5
ShH1.1b			36.6	39.7	43.5	30.5	48.5	25.2	22.4
ShH1.2				28.4	30.4	23.6	30.8	26.2	24.8
ShH1.3					30.7	34.6	40.4	28.1	24.0
ShH1.4						22.4	42.2	24.8	22.2
ShH1.5							28.2	24.4	15.4
EcH1.1								25.6	24.6
AtH1.1									27.6
HsH1.1									

It shows the homology of amino acids in the two proteins. Alignment in figure 2 was used for sequence identity analysis. Sh: *Sargassum horneri*, Ec: *Ectocarpus siliculosus*, At: *Arabidopsis thaliana* Hs: *Homo sapiens*
