# Supplementary material for: Sperm-specific histone H1 in highly condensed sperm nucleus of Sargassum horneri
Source: Sci Rep. 2024 Feb 9;14:3387. doi: 10.1038/s41598-024-53729-2 (PMC10858212; doi:10.1038/s41598-024-53729-2)
Supplement: Supplementary file 6 — Supplementary Table S2. [file 41598_2024_53729_MOESM6_ESM.pdf]

Supplementary Table S2. Prime list for PCR

Gene		Sequense(5'-3')	Predicted size (bp)
ShH1.1a	Forward	CCTGGCGCTCAACTTTACTC	193
	Reverse	TAGCCTTTTTGGGTGCAGAC	
ShH1.1b	Forward	GTCACGAAGAAAGCCGCTAC	181
	Reverse	ATGCCTTCTCAGCATTCGTT	
ShH1.2	Forward	GTCGTGGCCGGCAAGTTCATCA	134
	Reverse	GTCTTGCGGCTACCACCAGCAT	
ShH1.3	Forward	TGTTGACGCTTTGCTGGCCCT	141
	Reverse	CCGTTTTCGACCCCGAGCTTCA	
ShH1.4	Forward	AAGCACATCACCGCCACCCAT	146
	Reverse	TTCGGGGCGAGATTCATGGCCT	
ShH1.5	Forward	TGAGATGGTCGCTGAAGCTGTCG	127
	Reverse	AGCGCTTCGCAGCTGGTGAT	
ShMRP	Forward	GGCCACGGATATTGCGGGAAGA	143
	Reverse	TCGTTAGCGGCCGTCGGTGTTA	
ShH4	Forward	GTCGTGGCGGTGTGAAGCGTAT	135
	Reverse	TTGCGACGAGCGTGTTCGGT	
ShActin	Forward	CCCATCTACGAGGGTTACGC	107
	Reverse	GAGTAACCCCGCTCAGTGAG	
