# Supplementary material for: Sperm-specific histone H1 in highly condensed sperm nucleus of Sargassum horneri
Source: Sci Rep. 2024 Feb 9;14:3387. doi: 10.1038/s41598-024-53729-2 (PMC10858212; doi:10.1038/s41598-024-53729-2)
Supplement: Supplementary file 7 — Supplementary Table S3. [file 41598_2024_53729_MOESM7_ESM.pdf]

Supplementary Table S3. Detailed real-time PCR results.

ShH1.2

Template	Biological replicates	Sample types	Gene name	Ct	PCR efficiency	PCR efficiency^Ct	Ratio PCR efficiency^Ct (reference gene) /Ratio PCR efficiency^Ct (Target gene)	Melting temperature (°C)
Vegetative thallus	#1	Reference gene	Actin	18	2.08	531056	-	86.84
		Target gene	ShH1.2	23.4	2	11068835	0.05	87.01
	#2		Actin	17.61	2.08	399112	-	87.06
			ShH1.2	21.84	2	3754007	0.11	87.30
	#3		Actin	20.29	2.08	2841221	-	86.87
			ShH1.2	24.75	2	28215802	0.10	87.05
	#4		Actin	18.02	2.08	538891	-	86.93
			ShH1.2	22.47	2	5809570	0.09	87.10
	#5		Actin	16.82	2.08	223782	-	87.08
			ShH1.2	21.42	2	2805837	0.08	87.38
One (1N) nucleus stage conceptacle containing male receptacles	#1	Reference gene	Actin	21.36	2.08	6220608	-	86.73
		Target gene	ShH1.2	23.96	2	16318442	0.38	86.84
	#2		Actin	20.16	2.08	2583193	-	86.90
			ShH1.2	23.86	2	15225645	0.17	87.06
	#3		Actin	20.9	2.08	4441439	-	87.10
			ShH1.2	24.5	2	23726566	0.19	87.08
	#4		Actin	21.7	2.08	7979476	-	87.03
			ShH1.2	24.78	2	28808676	0.28	87.07
	#5		Actin	21.41	2.08	6452619	-	86.54
			ShH1.2	24.17	2	18875349	0.34	86.95
Sixty-four (64N) nuclei stage conceptacle containing male receptacles	#1		Actin	20.47	2.08	3241578	-	86.83
			ShH1.2	19.32	2	654485	4.95	87.10
	#2		Actin	20.51	2.08	3337944	-	87.01
			ShH1.2	18.57	2	389159	8.58	87.46
	#3		Actin	20.22	2.08	2699235	-	87.01
			ShH1.2	18.4	2	345901	7.80	87.27
	#4		Actin	20.17	2.08	2602181	-	86.94
			ShH1.2	22.22	2	4885247	0.53	87.27
	#5		Actin	20.54	2.08	3412093	-	86.80
			ShH1.2	19.25	2	623487	5.47	87.33

ShH1.3

Template	Biological replicates	Sample types	Gene name	Ct	PCR efficiency	PCR efficiency^Ct	Ratio PCR efficiency^Ct (reference gene) /Ratio PCR efficiency^Ct (Target gene)	Melting temperature (°C)
Vegetative thallus	#1	Reference gene	Actin	18	2.08	531056	-	86.84
		Target gene	ShH1.3	23.2	2.02	12138130	0.04	89.15
	#2		Actin	17.61	2.08	399112	-	87.06
			ShH1.3	21.57	2.02	3858594	0.10	89.30
	#3		Actin	20.29	2.08	2841221	-	86.87
			ShH1.3	24.79	2.02	37124486	0.08	89.18
	#4		Actin	18.02	2.08	538891	-	86.93
			ShH1.3	23.05	2.02	10923179	0.05	89.22
	#5		Actin	16.82	2.08	223782	-	87.08
			ShH1.3	21.62	2.02	3996655	0.06	89.38
One (1N) nucleus stage conceptacle containing male receptacles	#1	Reference gene	Actin	21.36	2.08	6220608	-	86.73
		Target gene	ShH1.3	25.11	2.02	46491428	0.13	89.006667
	#2		Actin	20.16	2.08	2583193	-	86.90
			ShH1.3	24.47	2.02	29644765	0.09	89.24
	#3		Actin	20.9	2.08	4441439	-	87.10
			ShH1.3	26.46	2.02	120114997	0.04	89.25
	#4		Actin	21.7	2.08	7979476	-	87.03
			ShH1.3	26.51	2.02	124412725	0.06	89.28
	#5		Actin	21.41	2.08	6452619	-	86.54
			ShH1.3	25.47	2.02	59882425	0.11	89.03
Sixty-four (64N) nuclei stage conceptacle containing male receptacles	#1		Actin	20.47	2.08	3241578	-	86.83
			ShH1.3	18.73	2.02	523879	6.19	89.13
	#2		Actin	20.51	2.08	3337944	-	87.01
			ShH1.3	18.31	2.02	389927	8.56	89.22
	#3		Actin	20.22	2.08	2699235	-	87.01
			ShH1.3	18.2	2.02	360907	7.48	89.17
	#4		Actin	20.17	2.08	2602181	-	86.94
			ShH1.3	23.46	2.02	14572804	0.18	89.21
	#5		Actin	20.54	2.08	3412093	-	86.80
			ShH1.3	19.46	2.02	875261	3.90	89.19

ShH1.4

Template	Biological replicates	Sample types	Gene name	Ct	PCR efficiency	PCR efficiency^Ct	Ratio PCR efficiency^Ct (reference gene) /Ratio PCR efficiency^Ct (Target gene)	Melting temperature (°C)
Vegetative thallus	#1	Reference gene	Actin	18	2.08	531056	-	86.84
		Target gene	ShH1.4	37.2	1.99	131019048896	0.0000041	86.91
	#2		Actin	17.61	2.08	399112	-	87.06
			ShH1.4	35.77	1.99	48975011109	0.0000081	85.56
	#3		Actin	20.29	2.08	2841221	-	86.87
			ShH1.4	33.83	1.99	12888416877	0.0002204	86.69
	#4		Actin	18.02	2.08	538891	-	86.93
			ShH1.4	37.25	1.99	135605435022	0.0000040	87.61
	#5		Actin	16.82	2.08	223782	-	87.08
			ShH1.4	33.21	1.99	8412220882	0.000027	85.76
One (1N) nucleus stage conceptacle containing male receptacles	#1	Reference gene	Actin	21.36	2.08	6220608	-	86.73
		Target gene	ShH1.4	32.8	1.99	6344259026	0.00098	85.51
	#2		Actin	20.16	2.08	2583193	-	86.90
			ShH1.4	30.14	1.99	1017259427	0.0025	85.55
	#3		Actin	20.9	2.08	4441439	-	87.10
			ShH1.4	34.89	1.99	26729069235	0.00017	85.68
	#4		Actin	21.7	2.08	7979476	-	87.03
			ShH1.4	36.41	1.99	76074746189	0.00010	87.28
	#5		Actin	21.41	2.08	6452619	-	86.54
			ShH1.4	35.3	1.99	35441622647	0.00018	87.36
Sixty-four (64N) nuclei stage conceptacle containing male receptacles	#1		Actin	20.47	2.08	3241578	-	86.83
			ShH1.4	20.8	1.99	1644917	1.97	85.58
	#2		Actin	20.51	2.08	3337944	-	87.01
			ShH1.4	20.65	1.99	1483597	2.25	85.62
	#3		Actin	20.22	2.08	2699235	-	87.01
			ShH1.4	20.63	1.99	1463319	1.84	85.62
	#4		Actin	20.17	2.08	2602181	-	86.94
			ShH1.4	28.48	1.99	324590359	0.01	85.90
	#5		Actin	20.54	2.08	3412093	-	86.80
			ShH1.4	22.15	1.99	4164814	0.82	87.33

ShH4

Template	Biological replicates	Sample types	Gene name	Ct	PCR efficiency	PCR efficiency^Ct	Ratio PCR efficiency^Ct (reference gene) /Ratio PCR efficiency^Ct (Target gene)	Melting temperature (°C)
Vegetative thallus	#1	Reference gene	Actin	18	2.08	531056	-	86.84
		Target gene	ShH4	23.36	1.93	4684020	0.11	83.66
	#2		Actin	17.61	2.08	399112	-	87.06
			ShH4	22.72	1.93	3075120	0.13	84.05
	#3		Actin	20.29	2.08	2841221	-	86.87
			ShH4	24.47	1.93	9718234	0.29	84.06
	#4		Actin	18.02	2.08	538891	-	86.93
			ShH4	23.43	1.93	4904647	0.11	83.59
	#5		Actin	16.82	2.08	223782	-	87.08
			ShH4	21.83	1.93	1712837	0.13	84.12
One (1N) nucleus stage conceptacle containing male receptacles	#1	Reference gene	Actin	21.36	2.08	6220608	-	86.73
		Target gene	ShH4	22.9	1.93	3461484	1.80	84.49
	#2		Actin	20.16	2.08	2583193	-	86.90
			ShH4	23.62	1.93	5557297	0.46	84.53
	#3		Actin	20.9	2.08	4441439	-	87.10
			ShH4	27.72	1.93	82347156	0.05	84.60
	#4		Actin	21.7	2.08	7979476	-	87.03
			ShH4	27.79	1.93	86225876	0.09	84.40
	#5		Actin	21.41	2.08	6452619	-	86.54
			ShH4	27.28	1.93	61659981	0.10	84.10
Sixty-four (64N) nuclei stage conceptacle containing male receptacles	#1		Actin	20.47	2.08	3241578	-	86.83
			ShH4	23.12	1.93	4000230	0.81	84.50
	#2		Actin	20.51	2.08	3337944	-	87.01
			ShH4	22.99	1.93	3672506	0.91	84.61
	#3		Actin	20.22	2.08	2699235	-	87.01
			ShH4	23.17	1.93	4133927	0.65	84.61
	#4		Actin	20.17	2.08	2602181	-	86.94
			ShH4	24.76	1.93	11759774	0.22	84.41
	#5		Actin	20.54	2.08	3412093	-	86.80
			ShH4	19.72	1.93	427750	7.98	84.65

Mastigoneme-related protein (MRP)

Template	Biological replicates	Sample types	Gene name	Ct	PCR efficiency	PCR efficiency^Ct	Ratio PCR efficiency^Ct (reference gene) /Ratio PCR efficiency^Ct (Target gene)	Melting temperatur e (°C)
Vegetative thallus	#1	Reference gene	Actin	18	2.08	531055.64	-	86.84
		Target gene	MRP	41.73	2.00	3647385935059.55	0.00000015	88.01
	#2		Actin	17.61	2.08	399112.50	-	87.06
			MRP	39.08	2.00	581101813183.12	0.00000069	87.29
	#3		Actin	20.29	2.08	2841221.33	-	86.87
			MRP	39.81	2.00	963838183561.95	0.0000029	88.24
	#4		Actin	18.02	2.08	538891.45	-	86.93
			MRP	38.27	2.00	331449931852.33	0.0000016	89.01
	#5		Actin	16.82	2.08	223782.07	-	87.08
			MRP	36.89	2.00	127349293525.86	0.0000018	89.29
One (1N) nucleus stage conceptacle containing male receptacles	#1	Reference gene	Actin	21.36	2.08	6220608	-	86.73
		Target gene	MRP	39.1	2.00	589213692200	0.000011	88.80
	#2		Actin	20.16	2.08	2583193	-	86.90
			MRP	35.32	2.00	42892311057	0.000060	90.63
	#3		Actin	20.9	2.08	4441439	-	87.10
			MRP	39.53	2.00	793808470081	0.000006	90.75
	#4		Actin	21.7	2.08	7979476	-	87.03
			MRP	42.46	2.00	6049696894147	0.0000013	88.24
	#5		Actin	20.05	2.08	2383251	-	86.86
			MRP	36.48	2.00	95846054932	0.000025	90.55
Sixty-four (64N) nuclei stage conceptacle containing male receptacles	#1		Actin	20.47	2.08	3241578	-	86.83
			MRP	21.09	2.00	2232146	1.45	90.66
	#2		Actin	20.51	2.08	3337944	-	87.01
			MRP	20.91	2.00	1970322	1.69	90.76
	#3		Actin	20.22	2.08	2699235	-	87.01
			MRP	20.46	2.00	1442360	1.87	90.69
	#4		Actin	20.17	2.08	2602181	-	86.94
			MRP	30.59	2.00	1616246388	0.0016	90.72
	#5		Actin	20.54	2.08	3412093	-	86.80
			MRP	21.78	2.00	3601084	0.95	90.69
