# Supplementary material for: Sperm-specific histone H1 in highly condensed sperm nucleus of Sargassum horneri
Source: Sci Rep. 2024 Feb 9;14:3387. doi: 10.1038/s41598-024-53729-2 (PMC10858212; doi:10.1038/s41598-024-53729-2)
Supplement: Supplementary file 8 — Supplementary Table S4. [file 41598_2024_53729_MOESM8_ESM.pdf]

Supplementary Table S4. Database of sequences used for phylogenetic trees (Figure 6).

Class	Order	Species	Data base									
Phaeophyceae	Dictyotales	*Dictyota dichotoma*	NCBI SRA	SRR5088952	SRR5088954	SRR5088955	SRR5088945					
	Ectocarpales	*Ectocarpus siliculosus*	accessionn number	CBJ32074	CBJ32082	CBJ33199	CBJ48888	CBN77002	CBN77455	CBN77460	CBN77561	
		Ectocarpus sp.	NCBI SRA	SRR1166429	SRR1166430	SRR1166441	SRR1660830	SRR5242551				
		Nemacystus decipiens onna1	gene ID	g13105.t1	g14808.t1	g7970.t1	g7977.t1	g7984.t1				
		Scytosiphon lomentaria	NCBI SRA	SRR5026364	SRR5026365	SRR5026634	SRR5026635	SRR6742571				
		Cladosiphon okamuranus S strain	gene ID	Cok_S_s046_7679	Cok_S_s098_10503	Cok_S_s101_10671	Cok_S_s156_12657	Cok_S_s165_13028				
	Laminariales	*Macrocystis integrifolia*	NCBI SRA	SRR3544557	SRR3615022							
		Macrocystis pyrifera	NCBI SRA	SRR5026588	SRR5026593	SRR5026594						
		Saccharina japonica	NCBI SRA	SRR5860560	SRR5860561	SRR5860563	SRR5860564	SRR5860564	SRR5860565	SRR5860566	SRR5860567	SRR5860568
		Undaria pinnatifida	gene ID	g01783.m1	g11632.m1	g11654.m1	g11660.m1	g11662.m1				
	Fucales	*Fucus ceranoides*	NCBI SRA	ERR1161611	ERR1161612							
		Fucus vesiculosus	NCBI SRA	SRR575725								
		Sargassum fusiforme	NCBI SRA	ERR2041176	SRR5357673							
		Sargassum hemiphyllum	NCBI SRA	ERR2041177	SRR5357786	SRR6161626						
		Sargassum henslowianum	NCBI SRA	ERR2041178								
		Sargassum integerrimum	NCBI SRA	ERR2041180	SRR5357667							
		Sargassum muticum	NCBI SRA	ERR2041183	SRR5357671							
		Sargassum thunbergii	NCBI SRA	ERR2041181	SRR826826							
		Sargassum vachellianum	NCBI SRA	ERR2041182								
		Sargassum vulgare	NCBI SRA	SRR3190990	SRR3190991	SRR3190992	SRR3190993					
		Sargassum horneri	NCBI SRA	ERR2041179	SRR5357783							
		Sargassum horneri	accessionn number	LC765405	LC765406	LC765407	LC765408	LC765409	LC765410			
	Ralfsiales	*Analipus japonicus*	accessionn number	LC765397	LC765398							
	Tilopteridales	*Mutimo cylindricus*	accessionn number	LC765399	LC765400	LC765401	LC765402					
	Desmarestiales	*Desmarestia aculeata*	accessionn number	LC765403	LC765404							
Bacillariophyceae	Naviculales	*Fistulifera solaris*	accessionn number	FiSOGAX10671	FiSOGAX12362	FiSOGAX21033	FiSOGAX22555					
		Phaeodactylum tricornutum	JGI Protin ID	33724	34960	44318						
	Thalassiosirales	*Thalassiosira pseudonana*	JGI Protin ID	264235	264244	264245	265086	268026	9992	9993		
		Thalassiosira oceanica	JGI Protin ID	100984	101092	74850	77133	90917	92300			
Oomycete	Peronosporaceae	*Phytophthora sojae*	accessionn number	XP_009517541	XP_009519138							
